# Machine Learning and Spatio Temporal Analysis for Assessing Ecological Impacts of the Billion Tree Afforestation Project

**DOI:** 10.1002/ece3.70736

**Published:** 2025-02-19

**Authors:** Kaleem Mehmood, Shoaib Ahmad Anees, Sultan Muhammad, Fahad Shahzad, Qijing Liu, Waseem Razzaq Khan, Mansour Shrahili, Mohammad Javed Ansari, Timothy Dube

**Affiliations:** ^1^ College of Forestry Beijing Forestry University Beijing China; ^2^ Key Laboratory for Silviculture and Conservation of Ministry of Education Beijing Forestry University Beijing China; ^3^ Institute of Forest Science University of Swat Swat Pakistan; ^4^ Department of Forestry The University of Agriculture Dera Ismail Khan Pakistan; ^5^ Precision Forestry Key Laboratory of Beijing Beijing Forestry University Beijing China; ^6^ Department of Forestry Science and Biodiversity, Faculty of Forestry and Environment Universiti Putra Malaysia Serdang Malaysia; ^7^ Department of Statistics and Operations Research, College of Science King Saud University Riyadh Saudi Arabia; ^8^ Department of Botany Hindu College Moradabad (Mahatma Jyotiba Phule Rohilkhand University Bareilly) Moradabad India; ^9^ Institute for Water Studies, Faculty of Science University of the Western Cape Cape Town South Africa

**Keywords:** afforestation, land‐use change, machine learning, NDVI, remote sensing, Sentinel‐2

## Abstract

This study evaluates the Billion Tree Afforestation Project (BTAP) in Pakistan's Khyber Pakhtunkhwa (KPK) province using remote sensing and machine learning. Applying Random Forest (RF) classification to Sentinel‐2 imagery, we observed an increase in tree cover from 25.02% in 2015 to 29.99% in 2023 and a decrease in barren land from 20.64% to 16.81%, with an accuracy above 85%. Hotspot and spatial clustering analyses revealed significant vegetation recovery, with high‐confidence hotspots rising from 36.76% to 42.56%. A predictive model for the Normalized Difference Vegetation Index (NDVI), supported by SHAP analysis, identified soil moisture and precipitation as primary drivers of vegetation growth, with the ANN model achieving an *R* ^2^ of 0.8556 and an RMSE of 0.0607 on the testing dataset. These results demonstrate the effectiveness of integrating machine learning with remote sensing as a framework to support data‐driven afforestation efforts and inform sustainable environmental management practices.

## Graphical abstract

This study evaluates Pakistan's Billion Tree Afforestation Project in KPK province using Random Forest classification and Sentinel‐2 imagery, revealing a 4.97% increase in tree cover from 2015 to 2023. Hotspot analysis showed increased vegetation clustering, while an ANN model predicted NDVI with high accuracy, identifying soil moisture and precipitation as key drivers of vegetation recovery. The research demonstrates the effectiveness of integrating machine learning with remote sensing for managing large‐scale afforestation projects, despite some areas remaining barren and requiring further intervention.

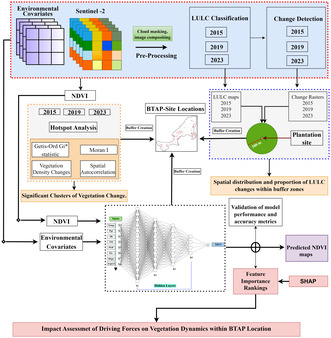

## 1 Introduction

Afforestation is important to global climate change mitigation, land rehabilitation, and biodiversity enhancement strategies. It has recently been announced that the BTAP Project in Pakistan is one of seven ambitious global initiatives and policy tools emphasizing scaling up forest landscape restoration (Kamal, Ali, and Yingjie 2018; Ullah et al. 2020). This afforestation activity removes large volumes of CO_2_ from the atmosphere, which is crucial in combating global warming (Haider et al. 2017; Jallat et al. 2021; Khan et al. 2020). It also enhances ecosystem services related to carbon sequestration and wildlife conservation (Chen and Zhang 2023; Wang et al. 2022; Wang et al. 2024). Remote sensing technologies have dramatically changed how afforestation programs are monitored and evaluated, predominantly through high‐resolution satellite imagery (Shawky et al. 2023; Zha et al. 2020). Such technological advancement now allows detailed global land observation, giving open access to imagery of all spatial scales from local to global (Galidaki et al. 2017; Zhang et al. 2024a, 2024b). The technology enables large‐scale, comprehensive analyses to increase the extent and resolution of studies to a new level (Colomina and Molina 2014; Huang et al. 2018).

Furthermore, when integrated with cloud computing platforms such as Google Earth Engine (GEE), giving advanced extensive data processing capabilities, remote sensing becomes an even more potent tool for the evaluation and monitoring of afforestation activities on a global scale (Anees et al. 2022a; Mehmood et al. 2024b). For instance, two panels of Landsat program‐derived, time series surface reflectance data from satellites (Fassnacht et al. 2019; Furniss et al. 2020), spanning four decades, have added much insight into land‐use and forest cover changes on the surface of the Earth (Smith et al. 2021). They are beneficial data to test afforestation and deforestation criteria and serve as an example of metrics in gauging the success or failure of projects like the BTAP. Advanced classification algorithms, such as RF, can successfully manage and analyze this vast reference database through platforms such as GEE (Cheng et al. 2022; Qasimi et al. 2023). RF is beneficial in this regard, as over the years, it has shown very high accuracy and, at the same time, meager rates of overfitting due to its ability to model intricate, non‐Gaussian relationships among variables (Ma et al. 2020; Mehmood et al. 2024e; Pang, Chang, and Chen 2022).

The Landsat program has been acquiring multispectral imagery for the last several decades (Irons, Dwyer, and Barsi 2012), which has led to spatially continuous and extensive data records of long‐term observations of land surface reflectance (Ouchra, Belangour, and Erraissi 2023; USGS 2022). Such records are essential for assessing changes in land use and modifications in the cover of forests from the past. They are irreplaceable in estimating the impacts of afforestation projects like the BTAP. This is a significant strength of the GEE platform: it can process and analyze this massive amount of data through advanced classification methods, such as RF. The RF algorithm, an up‐to‐date solution for better predictive power and generalization capacity, provides a good measure for preventing overfitting and increases accuracy in environmental assessments.

Remote sensing and machine learning have been increasingly highlighted in studies to monitor large‐scale afforestation, mainly through commercial plantations (Kupssinskü et al. 2020; Zheng, Abd‐elrahman, and Whitaker 2021; Anees et al. 2024a). The extensive integration of GEE and Landsat data has widely been applied to assess changes in forest cover and the sustainability of afforestation initiatives. Among different machine‐learning algorithms, RF and ANN performed well for land‐cover classification and afforestation monitoring (Mehmood et al. 2024a; Shahzad et al. 2024). RF has demonstrated high success in these types of studies at large scale and satisfactory accuracy, as it is typically able to separate well between different land‐cover types, with strong lines of evidence from numerous empirical case examples worldwide. In addition, the Random Forest's ability to model complex and nonlinearities in ecological data provides more efficient information for assessing afforestation programs (Hussain et al. 2024b).

In addition, combining remote sensing methods with biodiversity assessments has significantly increased biodiversity, particularly in degraded ecosystems (Zhang et al. 2024a, 2024b; Chen et al. 2023; Xie et al. 2023; Jiang et al. 2023). Evidence from numerous studies indicates that combining vegetation indices, such as NDVI, with machine‐learning models can effectively assess the ecological impacts of afforestation and support more strategic conservation efforts (Brieva et al. 2023; Mehmood et al. 2024c; Roy 2021; Anees et al. 2024a). Sustained, long‐term monitoring is increasingly recognized as crucial, as these processes often become observable only over decades. Scholars advocate for using temporal datasets to detect the frequently gradual or delayed impacts of afforestation initiatives, which are usually underestimated in short‐term studies (Nazir et al. 2019; Ullah et al. 2023; Zheng et al. 2024).

The applications of remote sensing to massive afforestation efforts face significant challenges and offer great opportunities, but there is a need for continuous innovation and complementary data sources. Coupling in situ validation with the integration of ecological models and remote sensing datasets enables long‐term prediction of ecosystem processes with a high level of reliability, together with an enhancement in accuracy when undertaking assessments (Doelman et al. 2020). Besides, proper monitoring of the effects of afforestation on the storage of soil carbon, diversity in microorganisms, and general health status of an ecosystem is essential in the provision of accurate information, as noted by studies focusing on the impacts of afforestation in different regions (Burke et al. 2023; Cao et al. 2011; Kong et al. 2022; Nave et al. 2013).

Approaches to integrating remote sensing data with biodiversity assessments have been demonstrated to improve our understanding of ecosystems massively (Bunce et al. 2014). Researchers have shown the efficacy of coupling vegetation indices, such as the NDVI, with machine‐learning methods for characterizing the ecological impacts of afforestation, thereby facilitating better conservation planning (Xiao, Xiao, and Sun 2020; Yao, Xiao, and Ma 2021; Anees et al. 2024a, 2024b). Regional‐based strategies benefit from more grounded policy support, while location specific investments are customized to local conditions (João et al. 2018).

The need for long‐term, consistent monitoring has gained recognition as necessary to capture the dynamic nature of forest ecosystems over time. Scholars propose the addition of temporal datasets to detect delayed or typically slow effects, often underestimated in brief‐term studies of initiatives like reafforestation (Hao et al. 2022; Zhang et al. 2022). Although implementing remote sensing within large‐scale afforestation projects is filled with limitations and potential, continuous change through multisensor data sourcing is essential. The coupling of ecological models with remote sensing data can provide improved predictions for long‐term outcomes, as this approach is validated by ground surveys, thereby increasing precision, reliability, and predictive accuracy (Pan, Harrou, and Sun 2023; Pan et al. 2023; Wang and Fan 2021).

While considerable progress has been made in applying remote sensing and machine learning to monitor afforestation efforts, existing work often lacks detailed long‐term examination of localized ecological outcomes (Anees et al. 2024a). Additionally, advanced techniques such as ANN and spatial analysis methods like hotspot analysis and Moran's *I* are not well exploited in evaluating vegetation dynamics associated with afforestation projects. This study aims to (1) review the ecological impact on land‐use and land‐cover (LULC) changes in response to the BTAP from 2015 to 2023, mainly focusing on rapid area changes using Sentinel‐2 imagery combined with the RF algorithm in the loess lands of Khyber Pakhtunkhwa (KPK) province. The research also measured major land‐cover type changes, such as tree cover, barren lands, and shrublands. (2) Conduct buffer analysis, hotspot analysis, and Moran's *I* spatial autocorrelation within the BTAP plantation buffer zones to study vegetation change over time. (3) Utilize machine‐learning methods, particularly ANNs, to forecast NDVI from biotic and abiotic factors, incorporating SHAP analysis to determine the primary drivers of vegetation change. This study explored how to effectively study large‐scale afforestation initiatives while drawing out key lessons applicable to forest cover and ecological restoration efforts more broadly.

## 2 Methods and Materials

### 2.1 Study Area

**FIGURE 1:**
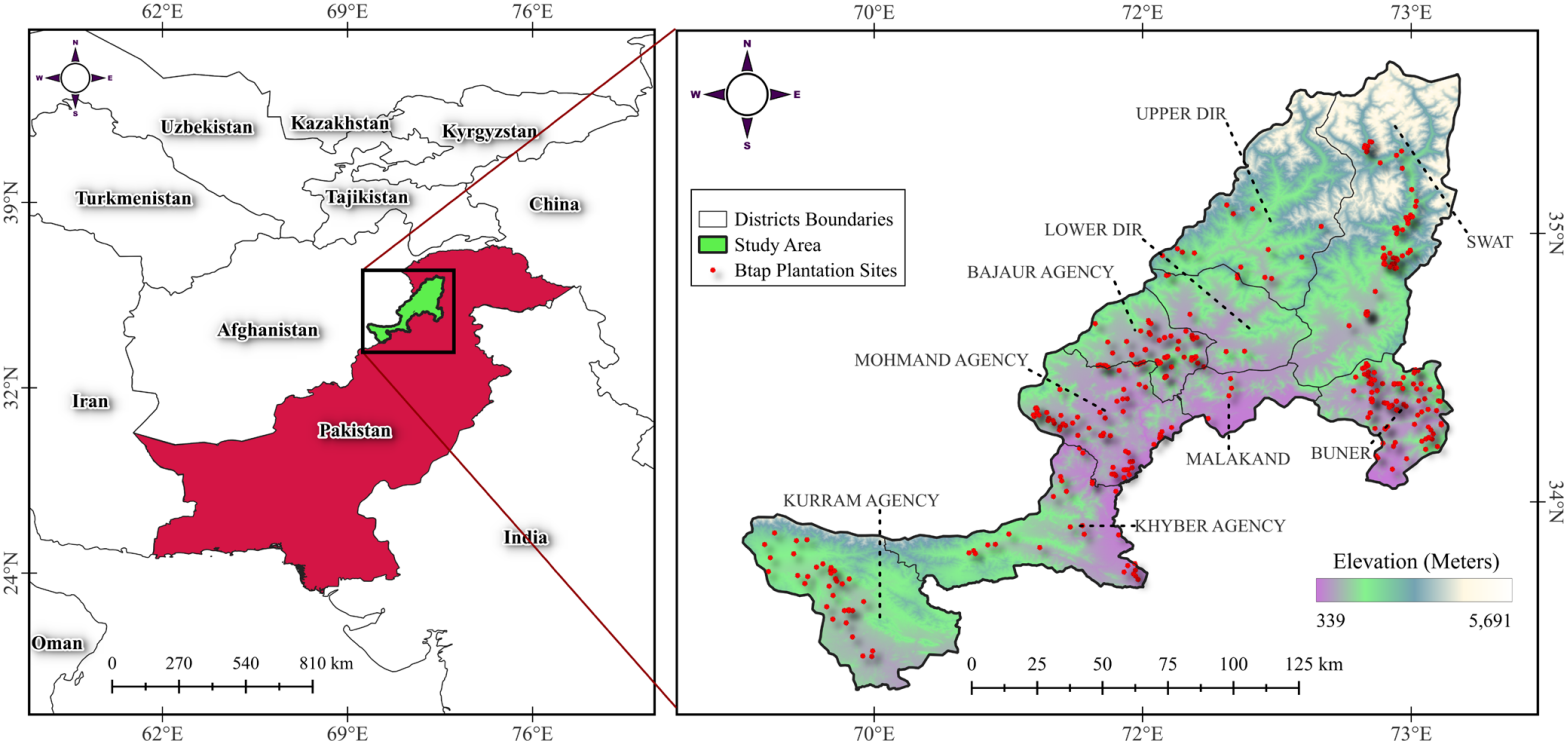
Map of the study area.

The study was conducted in the Khyber Pakhtunkhwa (KPK) province of Pakistan, located between latitudes 33.0°N and 36.0°N and longitudes 70.5°E and 73.5°E (Ahmed 2011; Mohiuddin 2007). KPK is a province with substantial geographic and ecological variation, making it an ideal location for the BTAP. Situated in the northwestern region of Pakistan, KPK is bordered by the Hindu Kush mountains to the north and the Indus River to the south, encompassing a range of climates from subtropical plains in the south to temperate and alpine zones in the north (Sohail et al. 2023) (Figure 1). The study focused on nine districts within KPK: Bajaur, Buner, Dir Lower, Dir Upper, Kalam, Khurum, Khyber, Malakand, and Mohmand. These districts were selected due to their critical role in the BTAP, which aims to rehabilitate degraded lands and enhance forest cover across the province.

The climate in KPK varies significantly across these districts. Southern regions, such as Buner and Khyber, experience hot summers and mild winters, while northern areas, including Kalam and Dir Upper, are characterized by cold winters with significant snowfall (Ali, Khan, and Ahmad 2018; Muhammad 2023). This climatic variability, combined with the province's complex topography, ranging from lowland plains to high mountain peaks, significantly influences the types of vegetation that thrive in the region (Bacha et al. 2021; Ul‐Haq et al. 2019; Anees et al. 2024b). This study leverages the diverse landscape and climate to investigate the effects of afforestation initiatives, like the BTAP, under different environmental conditions. The study sites were chosen based on their ecological significance, existing forest cover, and strategic importance within the BTAP framework. Additionally, the socioeconomic context of KPK, where local communities depend heavily on natural resources for their livelihoods, must be considered (Khan et al. 2019). The success of the BTAP is contingent on not only achieving ecological objectives but also promoting sustainable land‐use practices that benefit these communities.

### 2.2 Dataset and Preprocessing

Our study focuses on 344 plantation sites located in nine districts of KPK, Pakistan, which are strategically selected for the assessment of the BTAP. These sites are essential reference points for evaluating the project's impact on land‐cover changes. The geographical distribution of these plantation sites is categorized by district (Table 1).

**TABLE 1:** Distribution of plantation sites across districts in Khyber Pakhtunkhwa for the BTAP afforestation project (2015–2023).

S.No.	District	Number of plantation sites
1	Bajaur	57
2	Buner	84
3	Dir Lower (DirL)	5
4	Dir Upper (DirU)	15
5	Kalam	60
6	Khurum	34
7	Khyber	29
8	Malakand	4
9	Mohmand	56

This dataset offers an in‐depth view of the plantation sites, enabling a thorough evaluation of the BTAP and its impact on ecological restoration and sustainable land use across KPK. The spatial variability of the sites across different districts ensures a comprehensive assessment of the project's impact under diverse regional ecological and geographical conditions.

**FIGURE 2:**
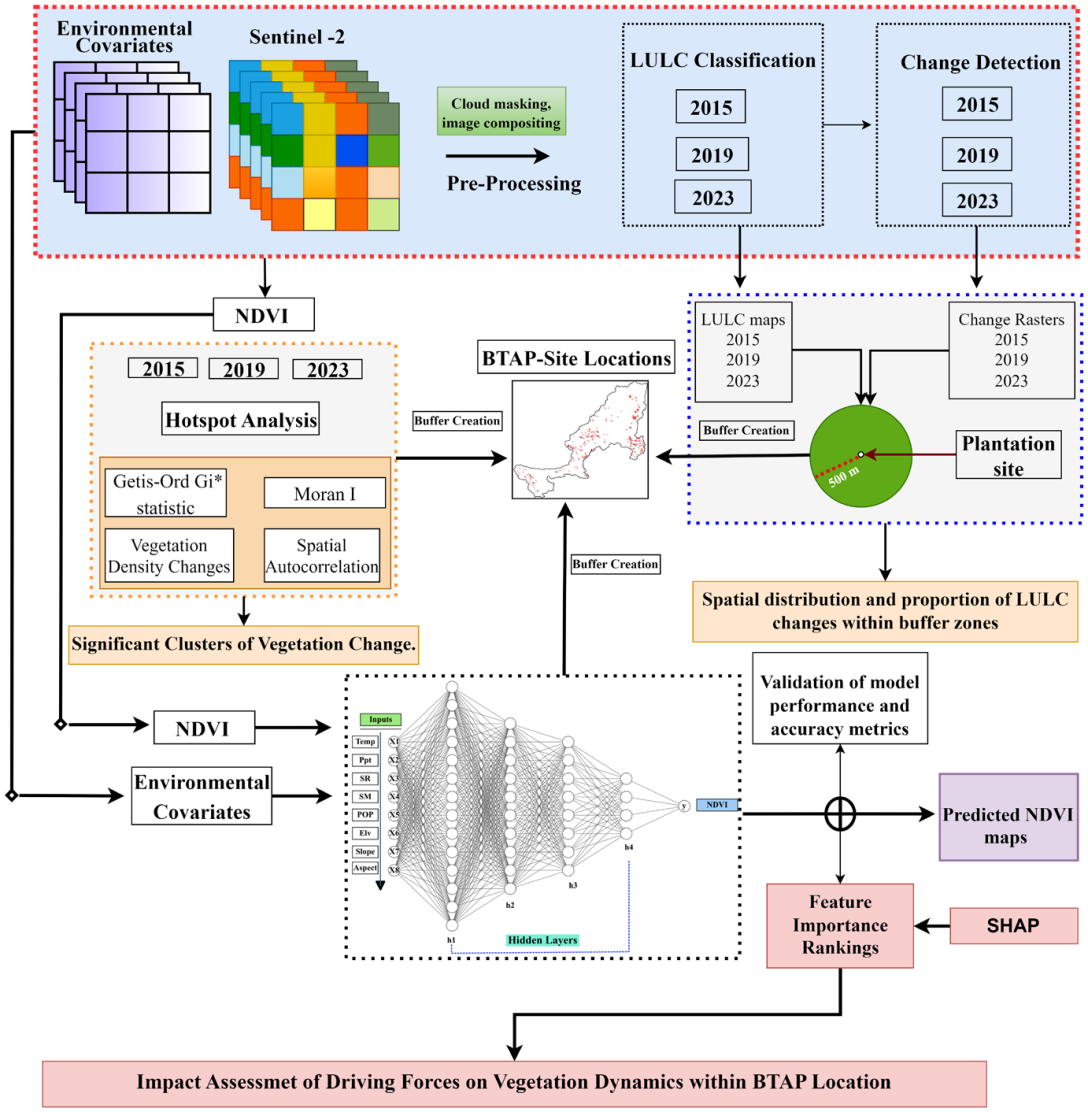
Schematic diagram of the research methodology.

This study used Sentinel‐2 satellite imagery to assess LULC changes from 2015 to 2023, specifically focusing on 2015, 2019, and 2023. The imagery was acquired for October and November, a post‐monsoon period characterized by significant vegetation growth and minimal cloud cover (Qiu et al. 2019; Valero et al. 2021). These periods were selected to exploit optimal conditions for clear, cloud‐free habitats necessary for accurate LULC mapping and vegetation assessment (Yan et al. 2022). Sentinel‐2 imagery was preprocessed in the GEE environment using the Sen2Cor algorithm to create cloud and shadow masks, ensuring the selection of cloud‐free pixels (Bui et al. 2022; Li et al. 2018; Xu, Li, and Chen 2022). To further enhance image quality, monthly composites for October and November were generated using median reflectance values, effectively mitigating residual clouds' impact or atmospheric disturbances (Castaldi et al. 2023; Rumora, Miler, and Medak 2020). Additionally, NDVI rasters for the respective years were derived from these processed images, providing a solid foundation for further analysis. The detailed schematic diagram of the research methodology is explained in Figure 2.

Buffer and autocorrelation analyses were performed to assess vegetation health and spatial distribution patterns around the plantation locations using these NDVI rasters as inputs. Alongside NDVI, a wide range of environmental variables, including temperature (temp), precipitation (Ppt), solar radiation (SR), soil moisture (SM), elevation, slope, aspect, and population density (POP) at the nearest second‐level administrative unit level to site locations, were considered as drivers for predictive NDVI models using machine‐learning approaches (Anees et al. 2024a) (Table 2). Utilizing the full‐scale computational capabilities of GEE, the processing workflow resulted in high‐quality, low‐cloud cover composites, ideally suited for LULC classification and change detection analyses. The carefully compiled datasets offer a strong and dependable foundation for assessing the ecological impacts of the BTAP plantation sites in Pakistan.

**TABLE 2:** Data sources, resolutions, and descriptions of environmental variables used in the study.

Data type	Source	Spatial resolution	Temporal resolution	Description	References
Sentinel‐2	European Space Agency (ESA)	10 m	5 days	Bands: Blue, green, red, NIR, SWIR	Tamiminia et al. (2024), Wang et al. (2019)
NDVI	ESA	10 m	5 days	NDVI derived from Sentinel‐2	Karlsen et al. (2021), Yang et al. (2022)
Temp	ERA5	9 km	Hourly/monthly	Surface temperature data	Gomis‐Cebolla et al. (2023), Muñoz‐Sabater et al. (2021)
Ppt	CHIRPS	5 km	Pentad	Climate Hazards Group InfraRed Precipitation with Station data	de Sousa et al. (2020), Vargas Godoy and Markonis (2023)
SR	TerraClimate: University of Idaho	4.5 m	Monthly	Downward surface shortwave radiation, used for climate and water balance studies	Abatzoglou et al. (2018), Abdi (2019), TerraClimate (2022), University of Idaho (2019)
SM				Soil moisture, derived using a one‐dimensional soil water balance model	
POP	World Pop	100 m	Annually	Gridded population density data	Gaughan et al. (2013), Stevens et al. (2015)
Topography (DEM)	ALOS World 3D (AW3D30)	30 m	—	Digital Elevation Model (DEM) data	JAXA (2019), Li and Zhao (2018)
Slope		30 m	—	Slope calculated from DEM	
Aspect		30 m	—	Aspect calculated from DEM	

### 2.3 LULC Classification Using the Random Forest Algorithm

To classify land use and land cover (LULC) for the years 2015, 2019, and 2023, we employed the Random Forest (RF) algorithm, a robust and widely recognized machine‐learning technique known for its exceptional accuracy in remote sensing applications (Badshah et al. 2024; Kumar and Agrawal 2023). The classification process was designed to categorize the landscape into seven distinct classes: Trees, Grassland, Swamp Vegetation, Arable Land, Shrubland, Built‐up Areas, and Barren Land. Training samples for each LULC class were meticulously collected from high‐resolution satellite imagery and validated against ground truth data where available (Hussain et al. 2024a). These samples were carefully distributed across the study area to ensure each class's balanced and representative dataset.

The spectral bands from Sentinel‐2 imagery, including visible, near‐infrared, and short‐wave infrared bands, were utilized as input features for the classification. In addition to these bands, vegetation indices such as the NDVI were calculated and incorporated to enhance the discrimination of vegetation related classes (Marino 2023; Marlina 2022; Anees et al. 2024b). The RF classifier was trained using thoroughly gathered training samples (Lange et al. 2017). The RF algorithm operates by constructing an ensemble of decision trees during the training phase, subsequently deriving its classification output based on the mode of the individual trees' predictions (Jun 2021). Key parameters of the RF classifier, including the number of trees and the “mtry” parameter, were systematically optimized to enhance overall classification accuracy. We used 500 decision trees, which optimized the balance between performance and computational efficiency. Additionally, the “mtry” parameter, controlling the number of variables available for splitting at each node, was tuned through a fivefold cross‐validation approach. This cross‐validation framework provided a robust evaluation process, ensuring that performance metrics were reliable and not biased by any single data partition. This parameter tuning was essential to ensure both model accuracy and stability (Anees et al. 2022b; Khan et al. 2024).

The refined RF model was subsequently applied to preprocessed Sentinel‐2 imagery for 2015, 2019, and 2023, producing classified LULC maps for each period. Each pixel was categorized into one of the seven LULC classes based on the model's predictions during this process. Postprocessing of these maps included general cleanup tasks like removing isolated pixels and smoothing out class boundaries to improve the overall consistency and accuracy of the classification results (Gupta, Kanga, and Mishra 2024; Wijaya, Munir, and Utama 2023). An independent set of validation samples was employed to evaluate classification accuracy. To ensure spatial independence and reduce potential overlap with the training data, these validation samples were selected from separate spatial regions. This spatial separation minimizes the risk of data leakage, providing a rigorous and unbiased assessment of model performance (Pomme et al. 2022). Feature importance scores were computed to identify the most influential input variables for LULC classification, with Sentinel‐2's near‐infrared and short‐wave infrared bands ranking among the highest. This analysis provides insight into the factors most critical for distinguishing between LULC classes and supporting model transparency.

To determine the BTAP's impact on land‐cover dynamics, a change detection analysis was performed using the LULC maps from 2015, 2019, and 2023. This analysis involved comparing the LULC maps from these time points, focusing on transitions to and from the “Trees” class, which indicate afforestation or deforestation activities (Vujović 2021). A change matrix was developed to quantify the transitions between different LULC classes over time, providing detailed insights into the extent and nature of land‐cover changes and identifying areas of significant change (Larbi 2023; Sadhwani, Eldho, and Karmakar 2023).

Spatial analysis was employed to map areas of significant land‐cover change, utilizing hotspot analysis and Moran's *I* autocorrelation to detect clusters of change and to evaluate the spatial patterns underlying these dynamics (Anees et al. 2020; Gomes et al. 2021). In addition, buffer analysis was conducted to examine LULC changes within the defined plantation buffer zones (Ziaul Hoque et al. 2022), thereby providing a more nuanced understanding of the localized impacts of the BTAP. The combined classification and change detection analyses yielded a comprehensive knowledge of LULC dynamics across Khyber Pakhtunkhwa, effectively elucidating the influence of the BTAP plantation sites over the study period. These analyses are pivotal in informing future conservation and afforestation strategies in the region, ensuring that efforts are targeted and effective.

### 2.4 Buffer Analysis

One of the objectives of this study was to assess the spatial–temporal variations in land‐cover types across various districts by analyzing land‐cover data within defined buffer zones around specific locations. This analysis was performed for 2015, 2019, and 2023 using high‐resolution classified LULC. A spatial point dataset containing particular locations within the districts was used to create buffer zones (Feng et al. 2021). A buffer radius of 500 m was selected to balance ecological and practical considerations, ensuring the inclusion of diverse land‐cover types within each buffer (Equation 1). A buffer distance of 500 m was selected for this study to capture the immediate ecological impact zone around the plantation sites (Chapagain et al. 2021; Yana and Rahayu 2017).

Preliminary analysis of multiple buffer sizes (100, 250, 500, and 1000 m) indicated that a 500‐m buffer provided the best balance between representing local landscape heterogeneity and capturing the spatial scale of plantation impacts (Horton et al. 2018; Liu et al. 2023). This distance is commonly used in vegetation and ecological studies to achieve a balance between detailed local analysis and broader generalization. A 500‐m buffer effectively includes the surrounding vegetation and land‐cover types likely influenced by plantation activities, as supported by previous studies using buffer ranges of 100 to 1000 m to assess afforestation impacts (Janeczko et al. 2019; Wibowo et al. 2015). Additionally, the 500‐m buffer maintains computational feasibility while providing meaningful insights into the direct influence of the plantation efforts.

(1) $$\text{Buffer zone area}=\pi \times {\left(500\;m\right)}^{2}==785,398\;m^{2}$$

#### 2.4.1 Spatial Analysis

Land‐cover data were extracted from the corresponding LULC for each buffer zone. This involved overlaying the buffer zones on the raster data and calculating the proportion of each land‐cover type within each buffer (Wang et al. 2024). The proportion of each land‐cover type within each buffer zone was computed (Equation 2)

(2) $${\text{Propostion}}_{i,j}=\frac{\text{Area of land}-\text{cover type}\;i\;\text{in buffer}\;j}{\text{Total area of buffer}\;j}$$

where *i* denotes the land‐cover type and *j* denotes the buffer zone.

The proportions of different land‐cover types were aggregated by district and year. This aggregation helped us to understand the overall land‐cover distribution and changes at the district level over time. The buffer analysis revealed significant improvements in land cover across various districts in KPK from 2015 to 2023, highlighting the success of the BTAP afforestation project.

### 2.5 Hotspot Analysis (Getis‐Ord Gi* Statistic)

In this study, we conducted a hotspot analysis to identify regions exhibiting significant variations in vegetation density. These regions display distinct positive or negative NDVI anomalies, indicating extensive spatial differences in vegetation. Utilizing remote sensing capabilities, we applied the Getis‐Ord Gi* statistic (Getis and Ord 2010, 1992; Ord and Getis 1995), a well‐established spatial statistical method, to pinpoint regions with significant vegetation changes. These regions, marked by notable NDVI values, show discernible positive or negative deviations in NDVI patterns. The Getis‐Ord Gi* statistic is crucial for locally identifying statistically significant spatial clusters characterized by high (hot‐spots) and low NDVI values (cold spots). (Equation 3).

(3) $$G_{I}^{*}\;\frac{\sum_{j=1}^{n}W_{i,j}X_{j}-\bar{X}\sum_{j-1}^{n}W_{i,j}}{\sqrt[{S}]{\frac{\left[n\sum_{j=1}^{n}W_{i,j}^{2}-{\left(\sum_{j=1}^{n}W_{i,j}\right)}^{2}\right]}{n-1}}}$$

wherein *x*_*j*_ delineates the attribute value associated with feature *j*: *w*_*i*_, *j* represents the spatial weight between features *i* and *j*, typically derived from their spatial relationship. *n* is the aggregate count of features.

Furthermore, the mean X and variance S of the attribute values are defined as Equations (4) and (5):

(4) $$\bar{X}=\frac{\sum_{j=1}^{n}W_{i,j}}{n}$$

(5) $$S=\sqrt{\frac{\sum_{J=1}^{N}X_{j}^{2}}{n}-{\left(\bar{x}\right)}^{2}}$$

The fundamental characteristic of the Gi* statistic is its ability to calculate the local aggregation of attribute values for a specific feature compared to its neighboring features, contrasting it with the aggregation across all features (Baldo et al. 2023). The presence of a statistically significant cluster is indicated by a notable departure from the expected local sum that exceeds the thresholds of random chance. This mathematical framework allows for a systematic and comprehensive investigation of geographical patterns, enabling researchers to accurately identify regions characterized by significant attribute concentration (Zhou et al. 2023).

Using ArcGIS Pro's Hotspot Analysis (Getis‐Ord Gi*) tool, we calculated spatial clustering metrics based on the tool's standardized procedures, which include automatic *z*‐score and *p*‐value calculations and corrections for spatial dependency using fixed distance bands. The Getis‐Ord Gi* statistic provides measurements of statistical significance for individual spatial features or regions. Two main metrics are calculated: the Gi* *p* value, which measures the likelihood, and the Gi* *z* score, which assesses the standard deviation (Garik 2021). The *z* score evaluates the level of concentration or dispersion within the features or regions. At the same time, the *p* value offers a probabilistic assessment to determine if the observed hotspot patterns could result from random spatial distributions. A substantial *z* score with a small *p* value indicates a statistically significant hotspot. In contrast, a significantly negative *z* score with a low *p* value indicates a statistically substantial cold spot. The size of the *z* score is directly proportional to the degree of clustering, with larger absolute values indicating more prominent clustering patterns (ESRI 2022, 2013).

Cold spot: Regions with a significant clustering of lower NDVI values, characterized by a Gi* *z* score less than −1.65. Hotspot: Areas showcasing a substantial aggregation of elevated NDVI values, marked by a Gi* *z* score greater than 1.65. Neutral areas: Regions that do not exhibit significant spatial correlation, falling within the *z* score range of −1.65 to 1.65. Confidence criteria were utilized to determine the statistical significance of the detected vegetation zones, with thresholds set at 90%, 95%, and 99%. Regions exhibiting pronounced Gi* *z* scores (either exceeding 2.58 or below −2.58) and minimal Gi* *p* values (< 0.01) are classified as “high confidence category,” signifying areas with extreme vegetation values at a 99% confidence level. The classification of vegetation zones based on confidence and significance levels is summarized in Table 3.

**TABLE 3:** Classification of vegetation hotspots and cold spots based on Getis‐Ord Gi statistic with corresponding confidence and significance levels*.

Classification	Confidence threshold	*p*	*z*
High‐confidence cold spots	99%	< 0.01	< −2.58
Moderate‐confidence cold spots	95%	< 0.05	< −1.96
Low‐confidence cold spots	90%	< 0.10	< −1.65
Nonsignificant	Not significant	N/A	−1.65 < *z* score < 1.65
Low‐confidence hotspots	90%	< 0.10	> 1.65
Moderate‐confidence hotspots	95%	< 0.05	> 1.96
High‐confidence hotspots	99%	< 0.01	> 2.58

Inverse distance weighted (IDW) interpolating the Getis‐Ord Gi* results generated raster maps of hot and cold spots aligned with Sentinel 2 resolutions (Sanusi et al. 2024). These were subsequently vectorized to produce polygonal representations of the vegetation anomalies. Following the identification and classification of vegetation hotspots and cold spots, the subsequent analysis focused on understanding the vegetation properties characteristic of these zones. This is critical due to the substantial contribution of vegetation to regional sustainability and the mitigation of environmental impacts.

### 2.6 Spatial Autocorrelation

To further evaluate the spatial autocorrelation of NDVI values within the buffer zones of the BTAP plantation sites for the years 2015, 2019, and 2023, we employed Moran's *I* statistic. This widely used measure for spatial autocorrelation in geographical data helps to understand the degree to which NDVI values are clustered or dispersed within the study area (Mielke et al. 2020). NDVI data points were obtained from a spatial dataset that included coordinates and NDVI values for each observation point within the BTAP buffer zones. Spatial weights were defined based on the k‐nearest neighbors' approach (Chen 2023).

Specifically, each observation was linked to its four nearest neighbors. This choice was informed by preliminary spatial analyses and validated through a sensitivity test over *k* = 3 to *k* = 8, which confirmed the robustness of observed spatial autocorrelation patterns. This was achieved using a k‐nearest neighbor algorithm, which identifies the eight closest points for each data point in the dataset (Okunev and Kushnareva 2023). The spatial weights matrix W was constructed such that *ω*_*ij*_ = 1 if locations *i* and *j* are neighbors and *ω*_*ij*_ = 0 otherwise. The weights were row standardized to ensure that the sum of weights for each row equals one, which normalizes the influence of neighbors (Chen 2021) (Equation 6).

(6) $$I=\frac{N}{S_{0}}∙\frac{\sum_{i=1}^{N}\sum_{j=1}^{N}\omega_{\mathrm{ij}}\left(x_{i}-\bar{x}\right)\left(x_{j}-\bar{x}\right)}{\sum_{i=1}^{N}{\left(x_{i}-\bar{x}\right)}^{2}}$$

where *N* is the number of spatial units indexed by *i* and *j*. $x_{i}$ is the NDVI value at location *i*. $\bar{x}$ represents the mean of the NDVI values. $\omega_{\mathrm{ij}}$ are the elements of the spatial weights' matrix W. $S_{0}=\sum_{i=1}^{N}\sum_{j=1}^{N}\omega_{\mathrm{ij}}$ Corresponds to the sum of all spatial weights.

The significance of Moran's *I* was tested using a randomization approach (Chen 2012). The null hypothesis posits no spatial autocorrelation (i.e., the observed spatial pattern is random). A low *p* value (typically less than 0.05) indicates significant spatial autocorrelation, confirming that the observed clustering or dispersion of NDVI values within the buffer zones is unlikely to be due to random chance (Gaspard, Kim, and Chun 2019). By integrating the Getis‐Ord Gi* hotspot analysis with Moran's *I* spatial autocorrelation, this study provides a comprehensive understanding of the spatial patterns and vegetation dynamics within the BTAP plantation buffer zones, highlighting areas of significant vegetation change and their spatial relationships.

### 2.7 Machine‐Learning Analysis for NDVI Prediction

This study employed ANN to predict NDVI based on a suite of climatic, topographic, and demographic variables. This method was chosen for capturing complex, nonlinear relationships within large datasets, making it ideal for ecological and environmental modeling tasks. The dataset included temp, Ppt, SR, SM, elevation, slope, aspect, and POP variables. These variables were carefully selected to reflect the key drivers of vegetation dynamics and served as predictors for NDVI, the response variable. The dataset was divided into training and evaluation subsets, with 70% allocated for model training and 30% reserved for model evaluation (Bradshaw et al. 2023; Gerber and Nychka 2021; Anees et al. 2024a; Luo et al. 2024). This split ensured that the model could be adequately trained while maintaining enough data to assess its predictive performance independently.

#### 2.7.1 ANN Model

**FIGURE 3:**
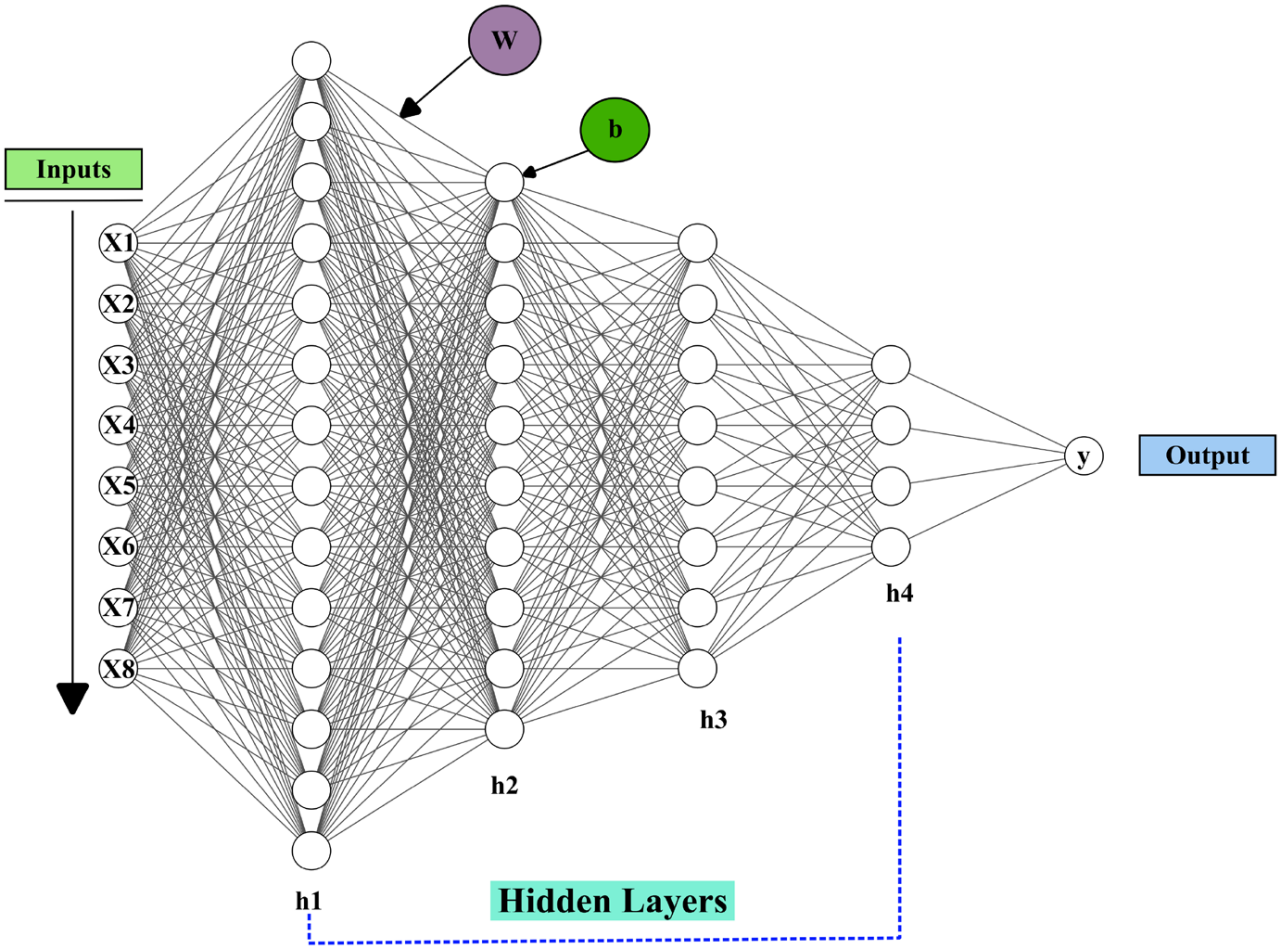
Schematic representation of a deep neural network used for regression analysis. The network consists of eight input features (X1 to X8), multiple hidden layers (h1–h4), and a single output node (y). Weights (w) and biases (b) are applied to each layer's connection. The hidden layers transform the input data through nonlinear activation functions, ultimately predicting the output value.

In this study, we implemented an ANN to predict NDVI using a comprehensive set of environmental and demographic variables. The ANN architecture consists of an input layer, four hidden layers, and an output layer inspired by the computational processes of the human brain (Lot et al. 2020; Madhiarasan and Louzazni 2022; Zhang et al. 2023; Huang et al. 2024; Zhang et al. 2022). To assess multicollinearity among predictors, we calculated the VIF for each variable, with all values provided in the supporting information (refer to Figure S2 and Table S1). To ensure robust model performance, a fivefold cross‐validation approach was employed during hyperparameter tuning. This method allowed us to assess model stability across different data splits, providing a more reliable estimate of predictive accuracy while minimizing the risk of overfitting. The decision to use four hidden layers was based on both empirical testing and existing literature, which suggests that deeper networks can capture complex, hierarchical patterns effectively, especially in ecological data modeling (Haq et al. 2022). Each hidden layer progressively reduced the number of neurons, 128 in the first layer, 64 in the second, 32 in the third, and 16 in the fourth, to enable the network to build increasingly abstract representations and reduce computational complexity without sacrificing performance (see Figure 3).

The ReLU (rectified linear unit) activation function was employed across all hidden layers, introducing nonlinearity into the model and enabling it to learn complex patterns in the data (Shahade et al. 2023). The Adam optimizer was chosen for its efficient handling of large datasets and adaptive learning rate, which facilitates faster convergence during training (Zhang 2019). The learning rate of 0.001, batch size of 64, and 200 epochs were selected based on extensive tuning and validation, as these values provided a balance between convergence speed and model accuracy. A systematic approach was adopted to tune the key hyperparameters, including the number of neurons in the hidden layers, the learning rate, and the number of epochs. This process was critical in refining the model's performance and ensuring accurate NDVI predictions. Table 4 outlines the final hyperparameters selected after extensive tuning, including a learning rate of 0.001, a batch size of 64, and an epoch count of 200.

**TABLE 4:** Hyperparameters used for training the ANN model.

Hyperparameter	Value
Number of hidden layers	4
Learning rate	0.001
Batch size	64
Number of epochs	200
Activation function	ReLU
Optimizer	Adam
Dropout rate	0.2
L2 regularization	0.01

To prevent overfitting, we incorporated dropout with a rate of 0.2 and L2 regularization with a strength of 0.01. Dropout randomly deactivates neurons during training, reducing the model's reliance on specific neurons and enhancing generalization (Ben Hamida et al. 2024). L2 regularization, which penalizes large weight magnitudes, was applied based on literature supporting its efficacy in improving model robustness, especially for regression tasks in environmental applications (Kim 2024). A validation set comprising 10% of the training data was also used to monitor performance and detect overfitting or underfitting. These hyperparameter choices, informed by empirical tuning and supported by relevant literature, enabled the model to achieve accurate NDVI predictions while maintaining generalization capabilities. To assess the uncertainty in model predictions, we conducted a Monte Carlo simulation involving 1000 iterations, where random noise was introduced to key predictor variables (e.g., temperature, precipitation, and elevation). This process generated a distribution of predicted NDVI values for each observation, reflecting the variability introduced by uncertainty in predictor measurements. We then calculated 95% confidence intervals (CI) for each prediction to quantify the expected range of true values. Furthermore, observations with high uncertainty (i.e., CI width exceeding a predefined threshold) were flagged to identify potential areas requiring additional data or model refinement.

#### 2.7.2 SHAP Feature Importance Analysis

We used SHapley Additive exPlanations (SHAP) as an interpretability framework to analyze the significance of input features in our NDVI prediction model. SHAP is model agnostic and provides a single measure of feature importance that can be used across all machine‐learning models (including ANN) (Younisse, Ahmad, and Abu Al‐Haija 2022). Traditional methods, such as feature importance in decision tree‐based models, are model specific. In contrast, SHAP values provide a general and theoretical approach to estimating the contributions of each feature. SHAP is based on game theory, and the SHAP value of a feature is what the model contributes to its output (Kim et al. 2023; Nordin et al. 2023). We average this contribution over all such combinations to provide an overall feature importance measure. The SHAP value for a given feature *j* is defined as (Equation 7):

(7) $$\varphi_{j}=\frac{1}{\left|N\right|!}\;\sum_{S\subseteq \text{Nleft}\left\{j\right\}}\left|S\right|!\left(\left|N\right|-\left|S\right|-1\right)!\left[f\left(S\cup \left\{J\right\}\right)-F\left(S\right)\right]$$

where *F* is the set of all features. *S* is any subset of the features *F* that does not include feature *j*. ∣*S*∣ is the number of features in subset *S*. *f*(*S*) is the prediction function applied to the subset *S*. *f*(*S*∪{*j*}) is the prediction function applied to the subset *S* with the addition of feature *j*. $\varphi_{j}$ represents the SHAP value for feature *j*. This formulation ensures that the SHAP value captures the average marginal contribution of a feature, considering all possible interactions with other features (Gebreyesus et al. 2023). The computation of SHAP values requires evaluating the model on different subsets of features, which can be computationally intensive but provides a robust measure of feature importance.

In our study, SHAP values were computed using the empirical approach implemented in the shapr package (Aas, Jullum, and Løland 2021; Kelemen et al. 2024), which ensures robustness in the estimation of feature contributions. After training the RF model to predict NDVI, an explainer object was created using the training dataset. The baseline prediction was set as the mean NDVI value of the training data. SHAP values were then calculated by evaluating the model's predictions across various subsets of input features from the evaluation dataset, simulating the contribution of each feature to the predicted NDVI. This analysis was performed in two stages: first, for the overall study area, and second, specifically for the plantation sites associated with the BTAP. SHAP values were calculated for each feature for the entire study area to understand their contribution to NDVI predictions across different environmental and topographic conditions in KPK. A separate SHAP analysis was conducted for the BTAP plantation sites to identify the key drivers of vegetation recovery within these afforestation zones. The SHAP values provided insights into the factors most critical in improving NDVI within these areas. The dual application of SHAP for the overall area and plantation sites provides a clear understanding of factors influencing vegetation dynamics, aiding informed decisions in managing and optimizing afforestation strategies in KPK.

### 2.8 Accuracy Assessment

The accuracy of both the RF and ANN models was rigorously evaluated using several statistical metrics: root mean square error (RMSE), mean absolute error (MAE), coefficient of determination (*R* ^2^), mean squared error (MSE), and the relative root mean square error (RRMSE%). These metrics were selected to assess the models' predictive performance across different dimensions comprehensively (Mehmood et al. 2024d; Xinde et al. 2023). The RMSE was used to quantify the average magnitude of the error between the predicted and actual NDVI values. As a metric sensitive to significant errors, RMSE provides valuable insights into the model's accuracy by measuring how well the model predicts NDVI across all data points (Doulah Md and Islam Md 2023; Pan, Harrou, and Sun 2023) (Equation 8).

(8) $$\text{RMSE}=\sqrt{\sum_{i=1}^{n}\frac{{\left(y_{i-}{\hat{y}}_{i}\right)}^{2}}{n}}$$

where *n* represents the number of observations, *y*_*i*_ the observed values, and $\hat{y}$ the predicted values.

The MAE was also calculated to complement RMSE. This metric measures the average absolute difference between predicted and actual values, providing a straightforward and easily interpretable assessment of prediction accuracy (Ağbulut, Gürel, and Biçen 2021; Nadakinamani et al. 2022; Guo et al. 2024; Zhang et al. 2024a, 2024b). Unlike RMSE, MAE treats all errors equally, making it useful for assessing the model's performance (Equation 9).

(9) $$\mathrm{MAE}=\frac{1}{n}\sum_{i=1}^{n}\frac{\left|y_{i}-{\hat{y}}_{i}\right|}{n}$$

The *R* ^2^ value, or the coefficient of determination, was used to determine the proportion of variance in NDVI that is predictable from the independent variables. This metric is beneficial for understanding the goodness of fit of the model, indicating how well the model captures the variability in the data (Nihar, Patel, and Danodia 2022; Suarez, Robson, and Brinkhoff 2023) (Equation 10).

(10) $$R^{2}=1-\frac{\sum_{i=1}^{n}{\left(y_{i}-{\hat{y}}_{i}\right)}^{2}}{\sum_{i=1}^{n}{\left(y_{i}-\bar{y}\right)}^{2}}$$

where $\bar{y}$ represents the mean of the observed values.

In addition, the MSE was computed, which represents the average of the squared differences between predicted and actual values (Equation 11), helps identify the variance of residuals, offering another perspective on the model's prediction errors (Ahmad et al. 2023).

(11) $$\mathrm{MSE}=\frac{1}{n}\sum_{i=1}^{n}{\left(y_{i}-\hat{y_{i}}\right)}^{2}$$

Finally, the RRMSE% was calculated to provide a normalized measure of prediction error relative to the mean observed value. RRMSE% (Equation 12), according to Shoko, Mutanga, and Dube (2018), is particularly useful in making the error magnitude more interpretable in the context of the scale of the data. This metric allows for an easier comparison across different datasets or studies.

(12) $$\text{RMSE}=\left(\frac{\text{RMSE}}{\bar{y}}\right)\times 100$$

By computing these metrics for the RF and ANN models, we ensured a robust and thorough evaluation of their predictive accuracy. This comprehensive approach allows for a detailed comparison between the models, highlighting their strengths and weaknesses in predicting NDVI based on the chosen environmental and demographic variables.

## 3 Results

### 3.1 LULC Classification and Change Detection Analysis

**FIGURE 4:**
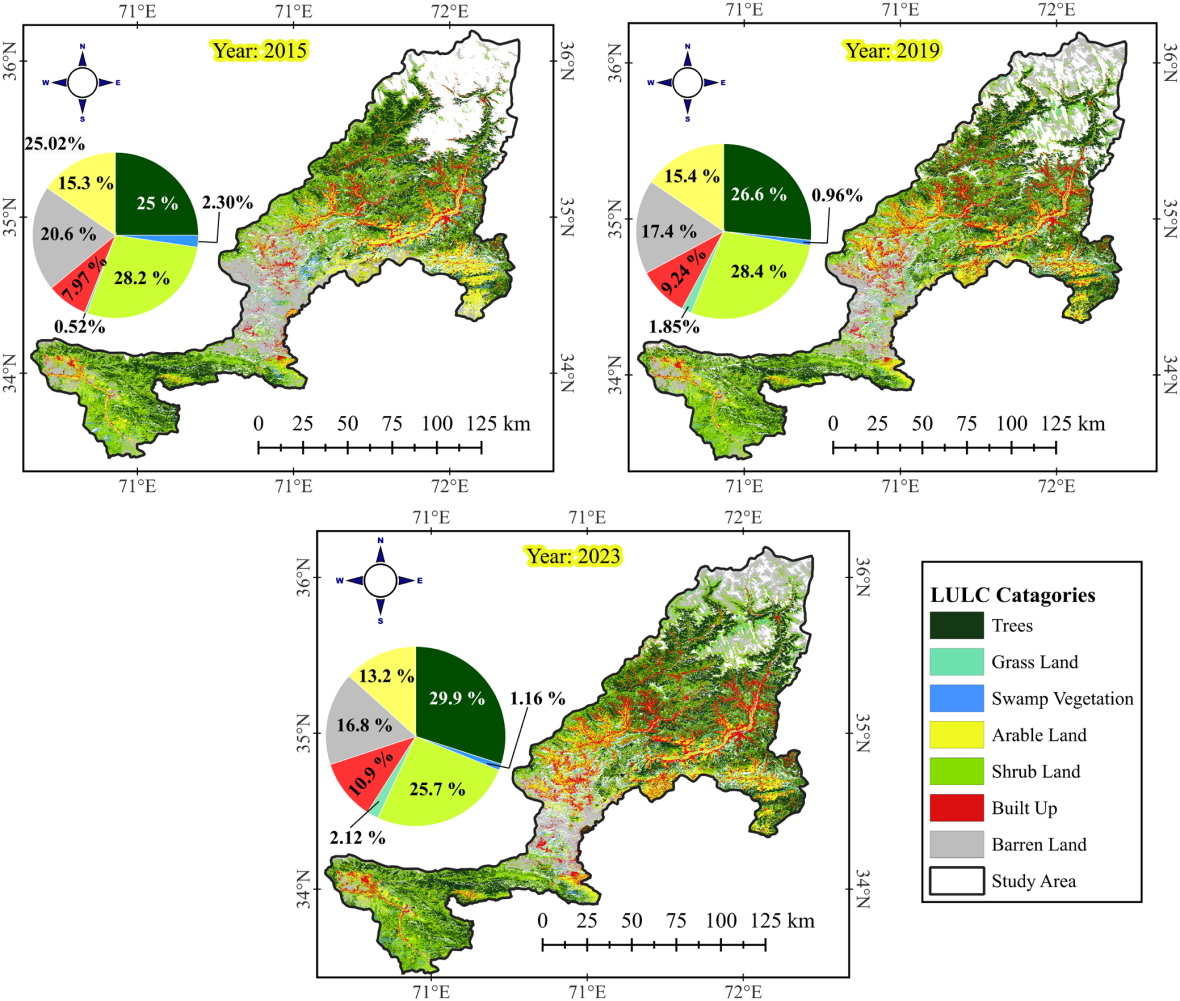
LULC classification maps for the years 2015, 2019, and 2023 within the study area, illustrating the spatial distribution and changes in LULC categories over time. The maps display eight LULC categories: Trees, Grassland, Swamp Vegetation, Arable Land, Shrubland, Built‐Up Areas, Barren Land, and the Study Area boundary. The accompanying pie charts show the percentage composition of each LULC category for each respective year, highlighting shifts in land‐cover types.

The LULC classification results for Khyber Pakhtunkhwa (KPK), Pakistan, across the years 2015, 2019, and 2023, reveal significant changes in land cover (Table 5). The area under tree cover increased from 5153.57 km^2^ (25.02%) in 2015 to 6835.66 km^2^ (29.99%) in 2023. Grassland areas expanded from 106.22 km^2^ (0.52%) in 2015 to 483.78 km^2^ (2.12%) in 2023. This growth suggests successful natural succession and practical afforestation efforts, contributing to improved ecosystem health. Arable land showed a slight fluctuation, increasing from 3164.76 km^2^ (15.36%) in 2015 to 3326.47 km^2^ (15.45%) in 2019 and then decreasing to 3024.83 km^2^ (13.27%) in 2023 (Figure 4). Built‐up areas expanded significantly from 1641.16 km^2^ (7.97%) in 2015 to 2483.85 km^2^ (10.90%) in 2023, reflecting urban growth and development. Barren land decreased from 4251.24 km^2^ (20.64%) in 2015 to 3830.70 km^2^ (16.81%) in 2023. This reduction indicates successful land rehabilitation and increased vegetation cover, further supporting the positive outcomes of the BTAP.

**TABLE 5:** LULC classification results for 2015, 2019, and 2023.

Class	Area (km^2^)	(%)	Area (km^2^)	(%)	Area (km^2^)	(%)
	2015		2019		2023	
Tree	5153.57	25.02	5729.32	26.62	6835.66	29.99
Grassland	106.22	0.52	398.17	1.85	483.78	2.12
Swamp vegetation	473.13	2.30	206.19	0.96	264.78	1.16
Arable land	3164.76	15.36	3326.47	15.45	3024.83	13.27
Shrubland	5811.06	28.21	6123.43	28.45	5866.51	25.74
Built up	1641.16	7.97	1989.04	9.24	2483.85	10.90
Barren land	4251.24	20.64	3752.29	17.43	3830.70	16.81

The change detection analysis for Khyber Pakhtunkhwa (KPK) between 2015 and 2023 provides valuable insights into the impacts of the BTAP, focusing on tree cover, shrubland, and barren land classes. Tree cover experienced significant growth throughout the study period. In the initial phase from 2015 to 2019, tree cover increased from 4983.2 km^2^ to 5898.8 km^2^, reflecting the early success of the afforestation efforts. This upward trend continued from 2019 to 2023, with tree cover expanding to 6835.66 km^2^. Tree cover saw a net increase of 5687.1 km^2^ over the entire period. The gains in tree cover amounted to 6352.1 km^2^, with a loss of only 665 km^2^, resulting in a 24.1% net change. These figures highlight the project's effectiveness in significantly enhancing forest cover in the region.

**FIGURE 5:**
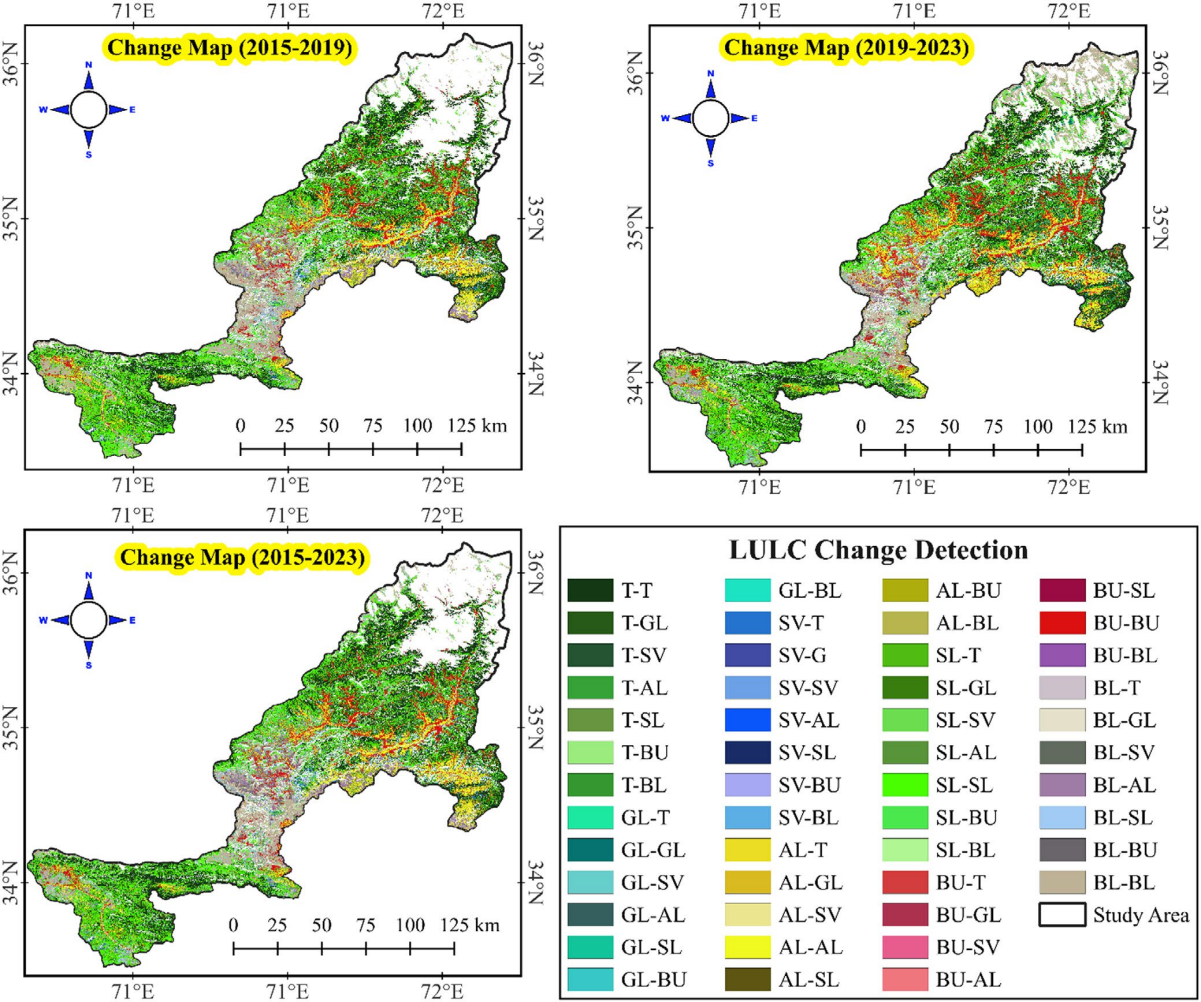
LULC change detection maps for the periods 2015–2019, 2019–2023, and 2015–2023, highlighting transitions between different LULC categories within the study area. Each color‐coded category in the legend represents a specific type of transition, such as forest‐to‐grassland, arable‐to‐built up, or shrubland‐to‐swamp, capturing the direction and nature of land‐cover changes over time. These maps reveal areas of significant land transformation, indicating patterns of urban expansion, deforestation, and vegetation shifts, which are essential for assessing the ecological impact of land‐use changes in the region.

Shrubland, a crucial vegetation type in the area, underwent notable changes, particularly transitioning to tree cover. From 2015 to 2019, about 475.3 km^2^ of shrubland was converted to tree cover. This trend persisted from 2019 to 2023, with an additional 857 km^2^ of shrubland transitioning to tree cover. Across the period, 889.2 km^2^ of shrubland was converted to tree cover (Figure 5). The net change in shrubland was 5232.7 km^2^, with 6928.9 km^2^ gained and 1696.2 km^2^ lost, resulting in a net change percentage of 22.1%. These transitions underscore the successful implementation of afforestation practices to increase forest cover while effectively managing shrub ecosystems. Barren land saw the most dramatic reduction, reflecting successful land restoration efforts by the BTAP. The area of barren land decreased from 2734.7 km^2^ in 2015 to 3022.7 km^2^ in 2019 and further to 2293.9 km^2^ in 2023. Over the entire study period, barren land was reduced by 1847.7 km^2^, with total gains of 3326.9 km^2^ and losses of 2154.8 km^2^, resulting in a net change of 1172.1 km^2^ and a net change percentage of 5%. This significant decrease in barren land highlights the project's effectiveness in converting degraded lands into productive vegetated areas, contributing to ecological restoration. The BTAP has markedly increased tree cover and effectively converted shrubland and barren land into forested areas, significantly improving the region's environmental health. The net increases in tree cover and the substantial reduction in barren land underscore the success of the BTAP's comprehensive approach to sustainable land use and ecological restoration in Khyber Pakhtunkhwa.

### 3.2 Buffer Analysis of BTAP Afforestation Project Within the Plantation Sites

**FIGURE 6:**
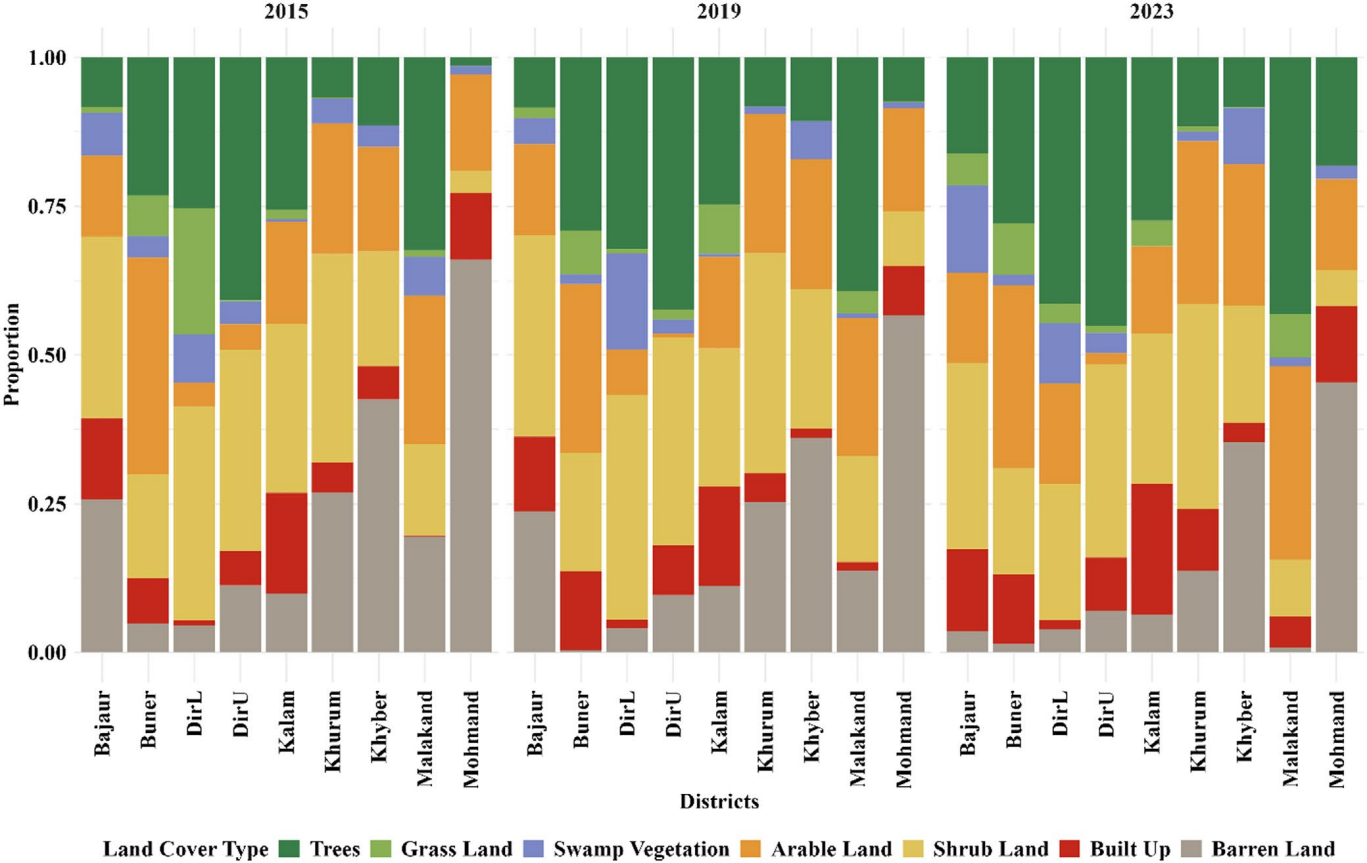
LULC changes in BTAP afforestation project buffer zones from 2015 to 2023. This figure illustrates the proportional changes in different LULC types across districts in the BTAP buffer zones over three time periods (2015, 2019, and 2023). The increase in tree cover (green bars) is evident across most districts, highlighting the success of afforestation efforts under the BTAP initiative. Concurrently, a decrease in barren land (brown bars) and built‐up areas (red bars) can be observed, signifying reduced land degradation and urban sprawl in some districts. Notable shifts in grassland (light green) and arable land (orange) proportions suggest varying impacts of land‐use policies and local ecological conditions across the districts. These results emphasize the regional variability in land‐cover changes and the critical role of afforestation in improving vegetation cover.

The BTAP afforestation project's buffer analysis reveals significant land‐cover improvements across various districts in Khyber Pakhtunkhwa (KPK) from 2015 to 2023. The project has successfully increased tree cover, a critical indicator of ecological restoration. For instance, tree cover in Bajaur expanded from 10.10% in 2015 to 19.20% in 2023, reflecting a remarkable 90.28% growth. This positive trend is reflected in other districts, such as Khyber, where tree cover rose from 8.25% to 14.75% over the same period, marking a 78.79% increase. Similarly, in Mohmand, tree cover increased from 9.87% to 18.33%, an 85.74% growth, indicating the effectiveness of the afforestation efforts across multiple regions (Figure 6). In addition to tree cover, changes in shrubland and other land‐cover types provide critical insights into the ecological impact of the afforestation project. Shrubland proportions have remained relatively stable in most districts, with slight variations. For example, in Bajaur, shrubland changed from 36.91% in 2015 to 37.22% in 2023, and in Khyber, it remained around 35%. In Mohmand, however, shrubland increased from 4.80% to 7.34%, suggesting some regions may be experiencing shifts in land‐cover types due to the project.

The status of afforestation, as indicated by the increased tree cover across all districts, is highly encouraging. However, the data also highlights potential areas for further plantation efforts. Grassland proportions, which have increased in many districts, indicate areas that may still be in transition and could benefit from additional afforestation activities. For instance, grassland in Bajaur rose from 1.11% in 2015 to 6.25% in 2023, while in Mohmand, similar increases were noted, suggesting these areas have potential for further forest cover expansion. Moreover, arable land, which showed minor changes, represents another opportunity for future afforestation. In Khyber, arable land remained stable, while Bajaur slightly decreased from 16.42% to 17.95%. These areas could be targeted for conversion to forested regions, thereby enhancing the overall ecological impact of the BTAP project. Barren land areas have shown noteworthy changes as well. For example, in Bajaur, barren land decreased from 11.35% in 2015 to 7.82% in 2023, indicating successful conversion to vegetated land. In Khyber, barren land also reduced from 9.51% to 6.48% during the same period. These decreases reflect the project's success in transforming previously barren areas into productive, vegetated land. Continued efforts to convert remaining barren regions into forested areas is crucial in sustaining the momentum of the BTAP project.

Overall, within the buffer zones of the plantation sites, significant land‐cover changes were observed from 2015 to 2023. In 2015, barren land accounted for 28.94% of the area, which reduced to 26.07% in 2019 and 17.48% in 2023 (Figure S1). Meanwhile, tree cover increased from 15.47% in 2015 to 19.20% in 2019, reaching 24.07% in 2023, indicating substantial afforestation progress. Additionally, shrubland proportions slightly decreased from 29.80% in 2015 to 25.79% in 2023. These changes reflect the effectiveness of the BTAP project in transforming barren lands into vegetated areas and enhancing overall ecological restoration.

#### 3.2.1 Analysis of LULC Transitions Within Plantation Buffer Zones

**FIGURE 7:**
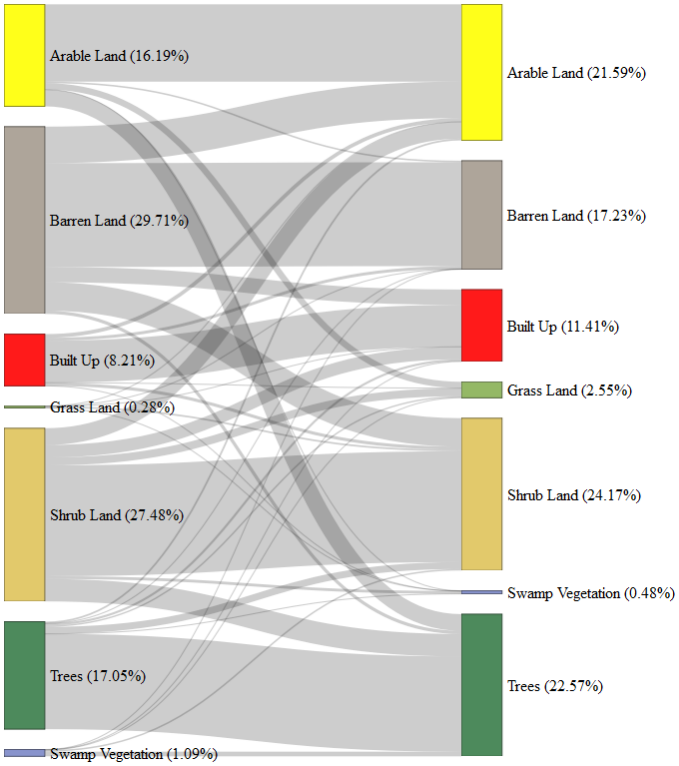
Sankey diagram illustrating the transitions between different LULC classes within 500‐m buffer zones around plantation sites from 2015 to 2023.

The analysis of LULC changes within 500‐m buffer zones around plantation sites reveals significant dynamics in land‐cover transitions, highlighting the impact of the BTAP. Notably, there were 16,057 transitions from barren to barren land, covering 33.4 km^2^, constituting 29.71% of all transitions (Figure 7). This indicates a high degree of persistence within barren land areas. However, significant transitions from barren land to other uses, such as 11.7 km^2^ to crops (17.23%) and 1.02 km^2^ to trees (22.57%), suggest a shift toward productive land‐use and potential ecological rehabilitation. The built‐up area experienced considerable changes, with 445,497 counts remaining built‐up (13.5 km^2^, 8.21%). There were notable transitions from built‐up areas to crops (1.19 km^2^, 16.19%) and shrubland (0.968 km^2^, 27.48%), reflecting ongoing urbanization pressures and the potential expansion of suburban areas. The stability of built‐up land cover shows continued urban and infrastructural development, impacting land‐use patterns.

Trees remained stable over 30.7 km^2^, accounting for 22.57% of the total transitions, suggesting effective preservation efforts and natural resilience. However, transitions from trees to shrubland (2.22 km^2^, 27.48%) and built‐up areas (0.676 km^2^, 8.21%) highlight urban expansion and infrastructure development pressures. Conversely, the transition from shrubland to trees amounted to 7.21 km^2^, indicating successful restoration and afforestation efforts to convert degraded lands into forested areas. This transition underscores the positive impact of BTAP in increasing tree cover within degraded regions. The dynamic nature of agricultural land use is evident, with 24.9 km^2^ of arable land (21.59%) remaining in crops, while transitions from crops to barren land accounted for 0.493 km^2^ or 16.19% of transitions. Such shifts reflect changes in agricultural practices or land abandonment. Notably, arable land also transitioned to tree cover over 5.37 km^2^, representing 22.57%, highlighting efforts to increase forest cover through afforestation.

The chi‐square test results, with a highly significant *p* value (< 0.001), suggest that the observed LULC changes are not random but influenced by underlying factors likely related to the impacts of plantation activities. Key insights include potential deforestation near plantation sites, as indicated by transitions from trees to shrubland and built‐up areas, highlighting pressures from urbanization and infrastructure development. Conversely, the significant transition from shrubland to trees underscores successful afforestation efforts to convert degraded lands into forested areas. Furthermore, the stability in certain land‐cover types, such as shrubland and tree cover, implies effective management practices or inherent resilience of these ecosystems.

### 3.3 Hotspot Analysis of NDVI Data Within the Buffer Zone of Plantation Sites (2015, 2019, and 2023)

**FIGURE 8:**
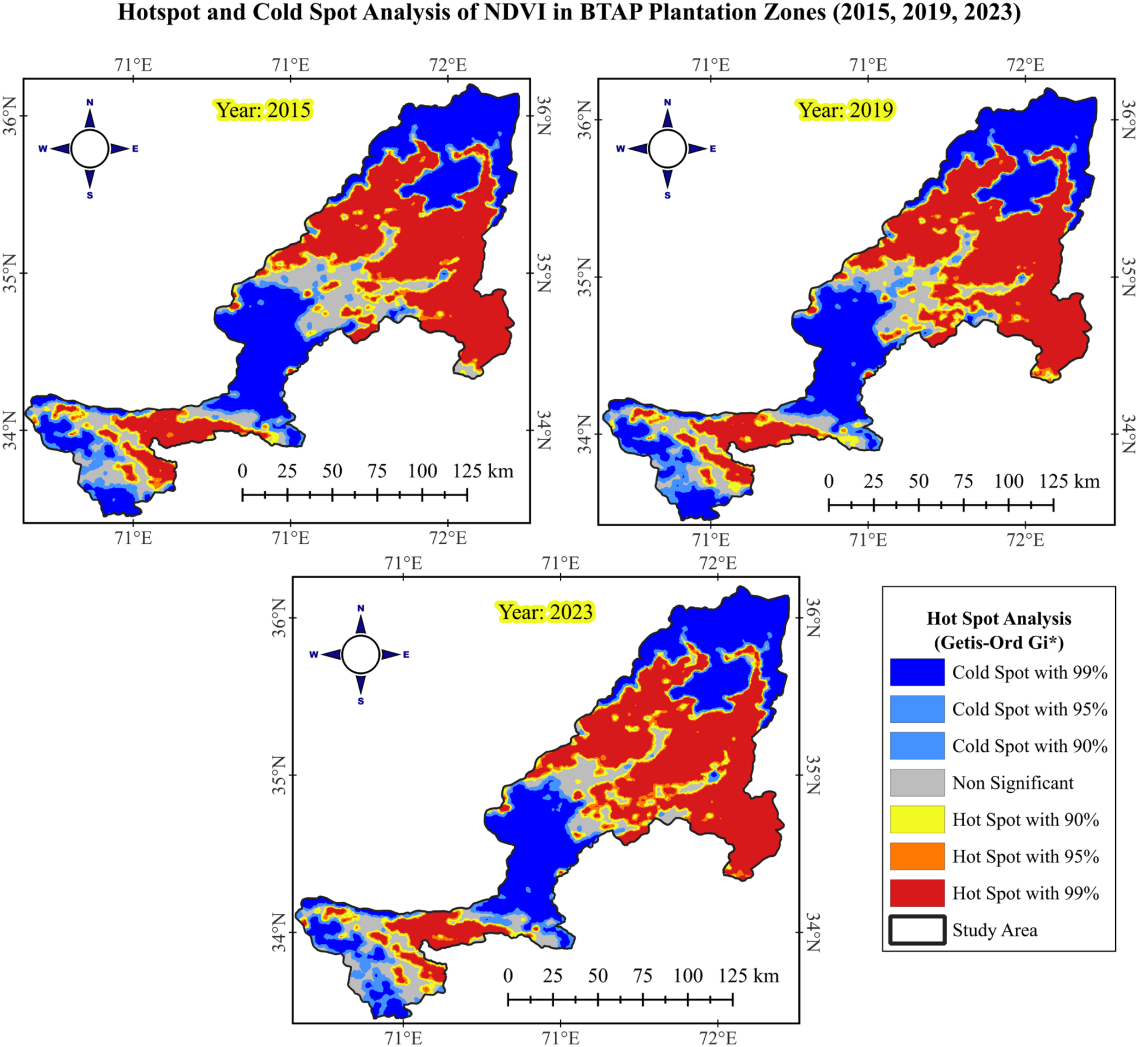
Spatial distribution of hotspots and cold spots in NDVI data within BTAP plantation buffer zones (2015, 2019, and 2023). This figure shows the spatial pattern of hotspots and cold spots of NDVI values within the BTAP plantation buffer zones for the years 2015, 2019, and 2023, using the Getis‐Ord Gi* analysis. Red areas represent statistically significant hotspots, indicating regions with consistently high NDVI values, while blue areas represent cold spots, indicating consistently low NDVI values. The spatial distribution of these areas provides insights into regions with high vegetation productivity and regions potentially affected by degradation or other stressors.

The hotspot analysis performed on the NDVI data for 2015, 2019, and 2023 provides significant insights into the spatial and temporal dynamics of vegetation in the study area. This section delves into the key findings, discussing the observed changes and their implications for the BTAP. In 2015, significant portions of the study region were classified as cold spots, particularly in the northern and central parts, indicating areas of lower vegetation density. By 2019, there was a noticeable increase in hotspots, especially in the southern and central regions, suggesting an improvement in vegetation density, likely due to the afforestation efforts under BTAP (Figure 8). This positive trend continued into 2023, with more areas in the northern region also showing high vegetation density, indicating a sustained impact of the afforestation project. The spatial distribution of hotspots and cold spots suggests that the southern areas have consistently improved vegetation density over the years, potentially due to targeted afforestation efforts. Initially, the northern regions displayed significant cold spots in 2015 but transitioned to include more hotspots by 2023, reflecting effective vegetation recovery efforts in these areas.

**FIGURE 9:**
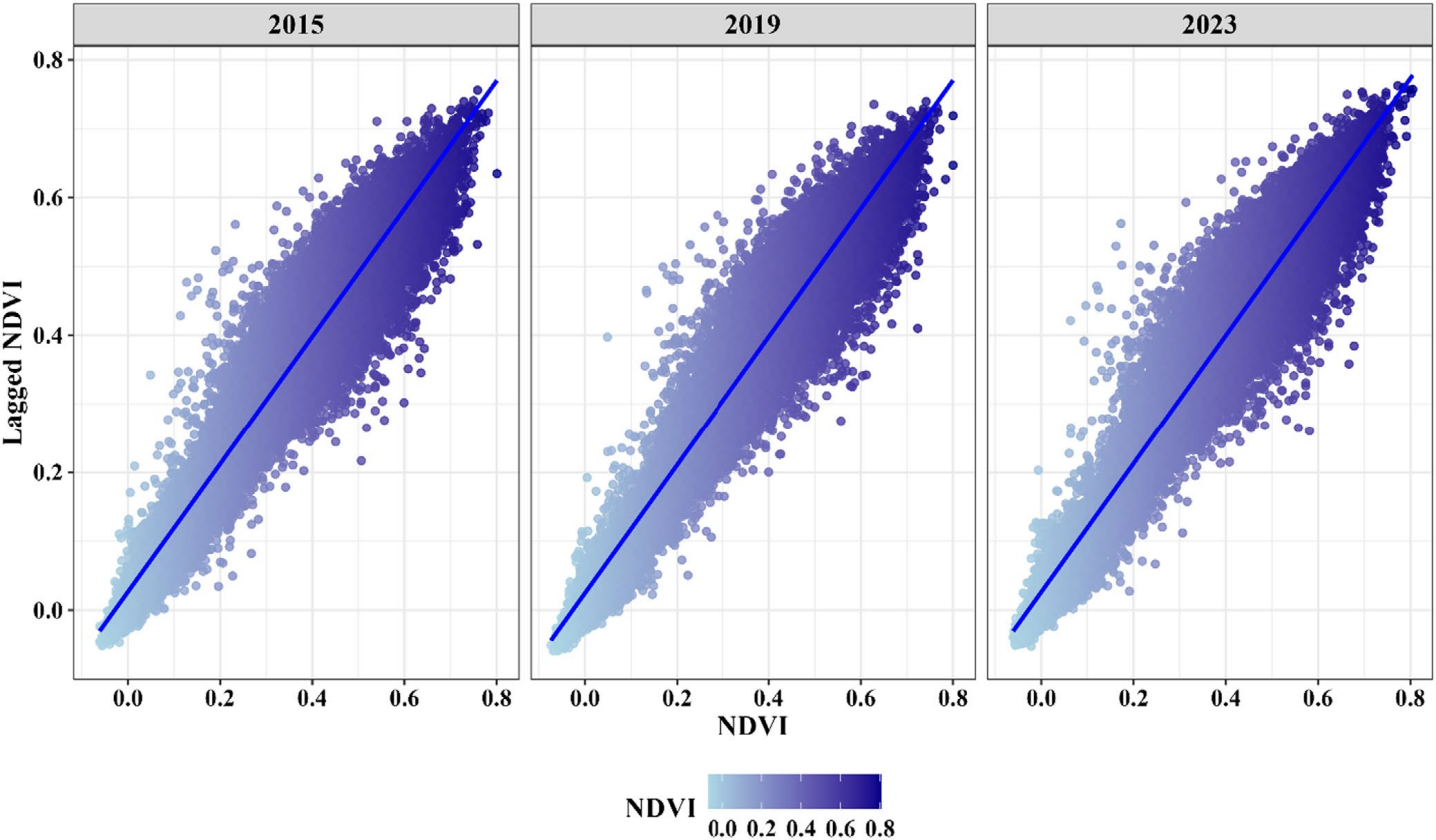
Moran's *I* scatterplots for NDVI values in 2015, 2019, and 2023 within BTAP plantation buffer zones.

The hotspot analysis revealed significant changes in vegetation density over the years. In 2015, 36.76% of the study area was identified as high‐confidence hotspots, while 28.03% were high‐confidence cold spots. By 2019, high‐confidence hotspots increased to 39.59%, and high‐confidence cold spots decreased to 25.44%. In 2023, high‐confidence hotspots rose to 42.56%, while high‐confidence cold spots reduced to 21.34%. Moderate‐ and low‐confidence hotspots and cold spots showed slight variations, reinforcing the positive impact of afforestation efforts (Table 6). In addition, Moran's *I* analysis for the years 2015, 2019, and 2023 revealed an increasing positive spatial autocorrelation, supporting the hotspot analysis findings. In 2015, the Moran's *I* value was 0.929, indicating strong clustering of NDVI values (*p* < 0.001). By 2019, Moran's *I* value slightly increased to 0.931, further clustering NDVI values (*p* < 0.001). In 2023, Moran's *I* value reached 0.933, suggesting an even stronger clustering of high NDVI values (*p* < 0.001) (Figure 9). The positive correlation further confirms the spatial clustering of NDVI, supporting the observed increase in vegetation density.

**TABLE 6:** Temporal hotspot analysis of NDVI data within BTAP plantation buffer zones (2015, 2019, and 2023).

Year	High‐confidence hotspots (%) (*n*)	Moderate‐confidence hotspots (%) (*n*)	Low‐confidence hotspots (%) (*n*)	Nonsignificant (%) (*n*)	Low‐confidence cold spots (%) (*n*)	Moderate‐confidence cold spots (%) (*n*)	High‐confidence cold spots (%) (*n*)
2015	36.76 (18,380)	5.90 (2950)	2.51 (1256)	23.09 (11,544)	1.43 (715 points)	2.50 (1250)	28.03 (14,015)
2019	39.59 (19,846)	5.44 (2720)	2.20 (1098)	22.29 (11,146)	2.21 (1104)	3.16 (1580)	25.44 (12,720)
2023	42.56 (21,168)	6.06 (3029)	2.26 (1128)	24.04 (12,008)	2.04 (1018)	2.94 (1468)	21.34 (10,668)

*Note: n* represents the Pixel counts.

### 3.4 Interannual NDVI Trends and Distribution Analysis

**FIGURE 10:**
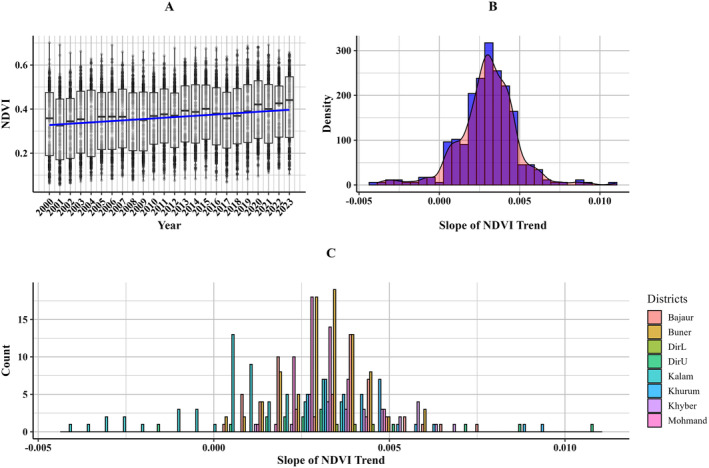
NDVI analysis across plantation sites. (A) Boxplot depicting the annual distribution of NDVI values from 2000 to 2023, with a trend line indicating the overall increase in vegetation greenness over time. (B) Histogram with density plot showing the distribution of the slope of NDVI trends across all plantation sites, highlighting the generally positive trend in NDVI. (C) Histogram illustrating the count of NDVI trend slopes for different districts, with each color representing a specific district, indicating variability in vegetation trends across the study area.

This study examines the interannual trends of NDVI across 343 plantation sites involved in the Billion Tree Afforestation Project (BTAP). Utilizing linear regression, the analysis offers insights into temporal changes in NDVI, thereby reflecting the ecological impacts of the afforestation efforts. The results indicate a statistically significant positive trend in NDVI across all plantation sites, with an estimated slope of 0.0030 (*p* < 0.01), signifying a consistent annual increase in vegetation density. This trend underscores the success of the BTAP in enhancing vegetation cover and improving ecosystem health at these plantation sites (Figure 10A).

The density plot of NDVI slopes reveals a right‐skewed distribution, suggesting that while most sites experienced moderate improvements, a subset achieved exceptionally high growth rates (Figure 10B). The peak of this distribution centers around a slope of approximately 0.003, further corroborating the overall positive trend. Notably, many sites exhibit slopes clustered near zero, indicating stable NDVI values with minimal change over time (Figure 10C). These observed variations may be influenced by local environmental conditions, management practices, or species selection, underscoring the need for tailored afforestation strategies to optimize outcomes across diverse ecological contexts.

Among the districts analyzed, Bajaur and Mansehra demonstrated the most considerable NDVI improvement, with average slopes of 0.0063 and 0.0061, respectively, reflecting highly effective afforestation efforts. Abbottabad and Swat showed intermediate performance, with slopes of 0.0040 and 0.0038, suggesting moderate success that could benefit from enhanced management strategies. Conversely, Lower Dir exhibited the lowest improvement, with an average slope of 0.0015, highlighting significant challenges in this region.

### 3.5 Model Performance and Validation

**FIGURE 11:**
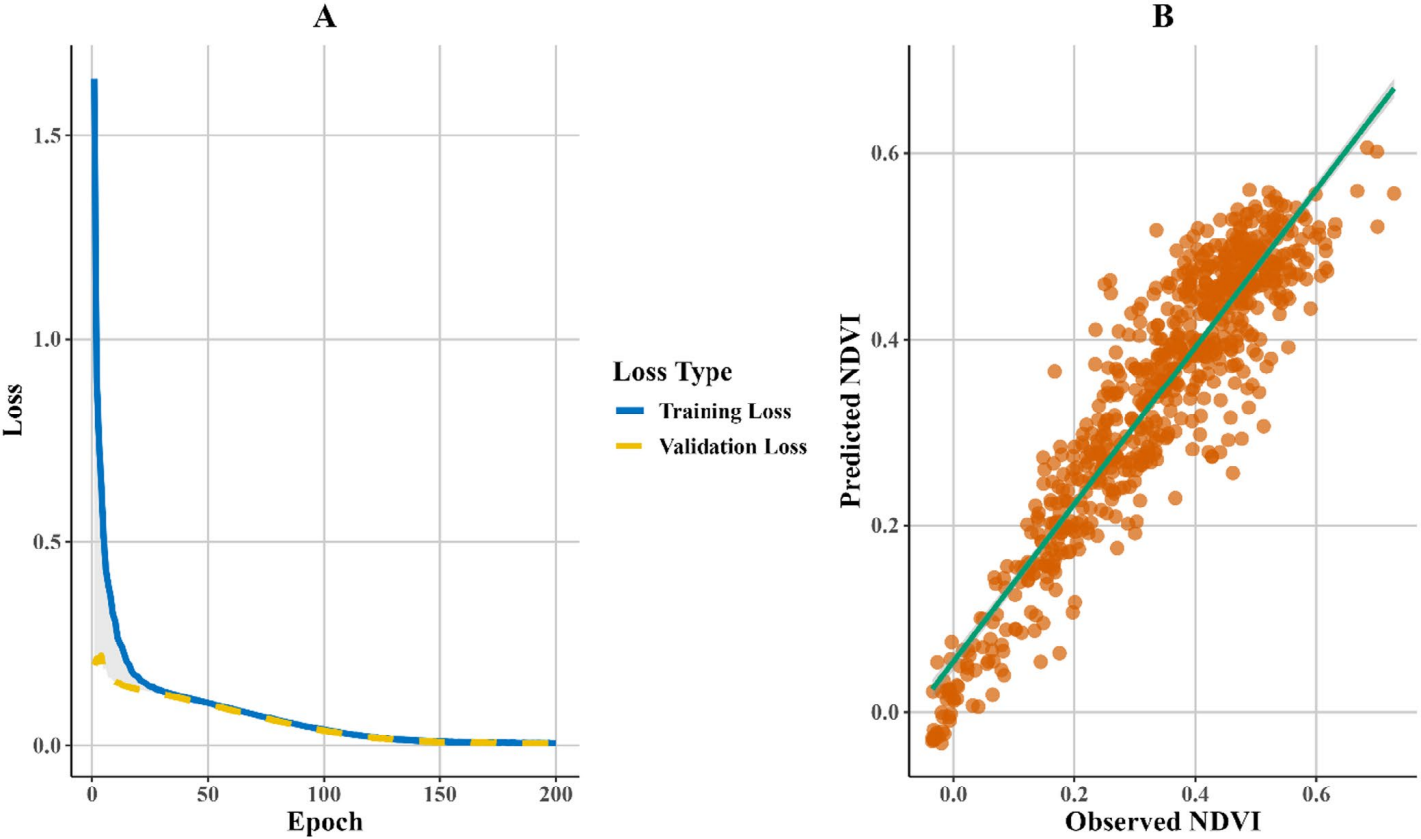
(A) Training and validation losses of the ANN model. (B) Scatter plot of predicted versus observed NDVI values.

The ANN model's architecture, optimized through careful hyperparameter tuning, was subjected to training and validation to evaluate its predictive performance. The training process spanned 200 epochs, which allowed the model to converge effectively without imposing excessive computational demands (Figure 11A), the training and validation loss curves converged smoothly, indicating that the model learned effectively from the data without overfitting. This result underscores the success of the chosen hyperparameters in balancing model complexity with generalization ability.

Further evaluation of the model's performance is depicted in Figure 11B, which shows a scatter plot comparing the predicted NDVI values against the observed NDVI values. The strong alignment of the data points along the diagonal line suggests a high degree of accuracy in the model's predictions, with minimal deviation from the actual NDVI values. The *R* ^2^ value of 0.8556 further confirms that the model explains approximately 85.56% of the variance in the observed NDVI values. Additionally, the RMSE of 0.0607 indicates a low average prediction error, further validating the model's robustness (Table 7). The testing phase of the model also demonstrated strong performance metrics. The MSE was 0.0037, with an RMSE of 0.0607 and an *R* ^2^ value of 0.8556. The RRMSE% was calculated at 17.87%, and the MAE was 0.0461, reflecting the model's accuracy in predicting NDVI values based on the input variables. The validation phase involved testing the model on new sites of plantation across three districts (Mardan, Charsada, and Peshawar), outside the area where the model was trained. This external validation highlights the model's ability to generalize to unseen data. The validation results include an MSE of 0.0057, RMSE of 0.0758, and an *R* ^2^ value of 0.7818, indicating that the model explains approximately 78.18% of the variance in the observed NDVI values (Figure S4). The MAE of 0.0606 and RMSE% of 21.80% suggest a moderate level of accuracy when applied to these new sites, demonstrating the model's practical utility in predicting NDVI for regions outside the training area.

**TABLE 7:** Performance metrics of the ANN model.

Metric	Training data	Testing data	Validation data (new sites)
MSE	0.0006	0.0037	0.0057
RMSE	0.0255	0.0607	0.0758
*R* ^2^	0.8765	0.8556	0.7818
MAE	0.0191	0.0461	0.0606
RMSE%	7.42%	17.87%	21.80%

In addition to the testing results, the training data exhibited strong performance, indicating that the model was well‐tuned during the training phase. The training data results are as follows: MSE of 0.0006, RMSE of 0.0255, and an *R* ^2^ value of 0.8765. The MAE for the training data was 0.0191, indicating the model's effectiveness in learning from the training dataset. Additionally, the RRMSE% for the training data was calculated to be 7.42%, which reflects the model's accuracy relative to the average magnitude of the observed NDVI values. The systematic selection of hyperparameters, combined with regularization and continuous monitoring during training, resulted in a strong model capable of accurately predicting NDVI across diverse environmental conditions. The selected hyperparameters minimized the validation loss, ensuring the model could generalize well to unseen data.

**FIGURE 12:**
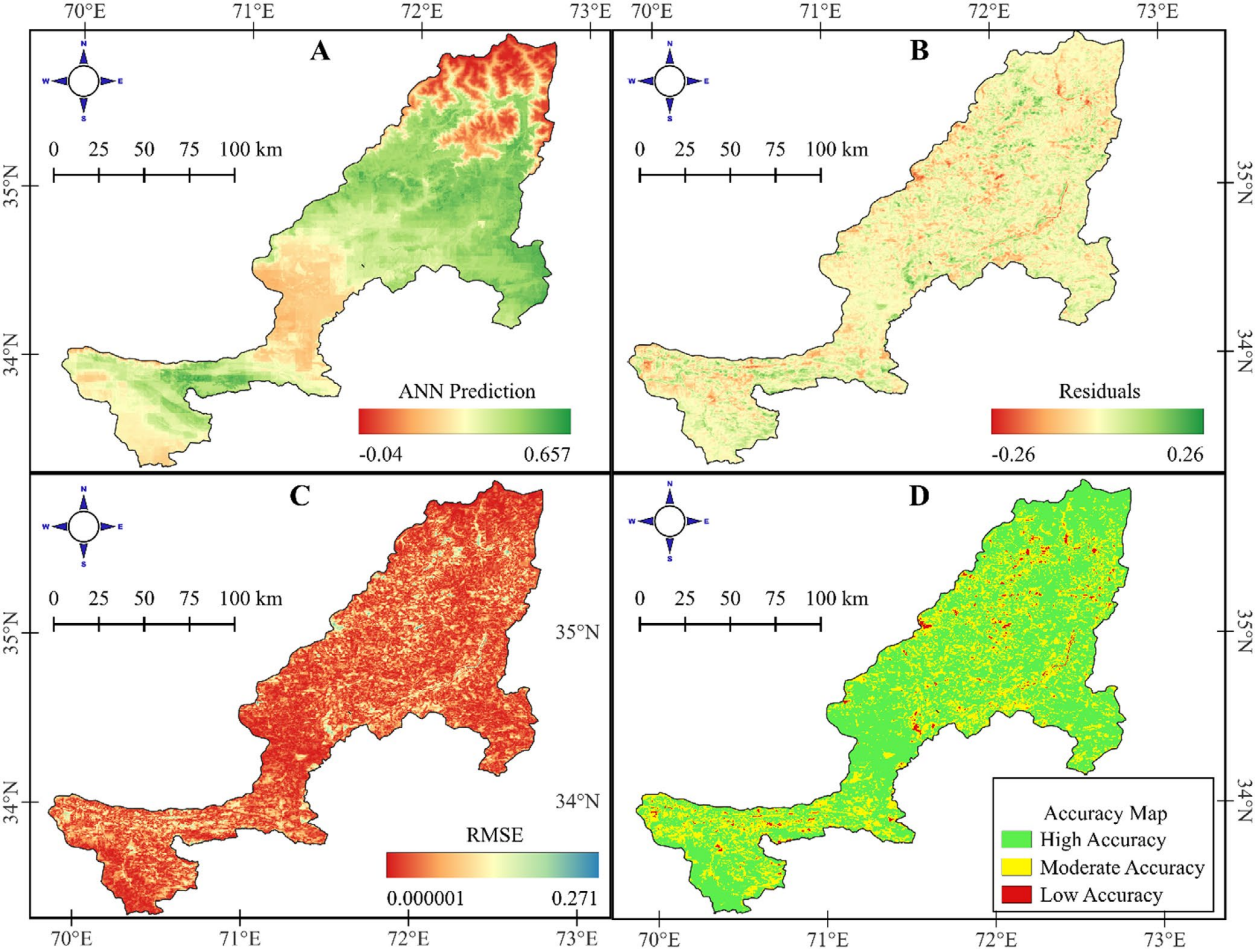
Spatial analysis of NDVI prediction accuracy using an ANN model. (A) ANN prediction map displaying predicted NDVI values across the study area. (B) Residual map illustrating the difference between observed and predicted NDVI values, highlighting areas of over‐ and underestimation. (C) RMSE map shows the RMSE of the predictions, with lower values indicating better model performance. (D) Accuracy map categorizing the study area into zones of high, moderate, and low accuracy based on the residuals and RMSE, providing insight into the model's performance across different regions.

The model's spatial predictions of NDVI, shown in the predicted NDVI raster Figure 12A, provide a detailed distribution of predicted values across the study area, ranging from −0.0462 to 0.6349. Residual and RMSE rasters were generated to assess spatial accuracy. The residual raster Figure 12B displays prediction errors, with values from −0.2713 to 0.2672, indicating areas of underestimation and overestimation. The RMSE raster Figure 12C shows average prediction errors, with values from 0.0000007 to 0.2713, highlighting regions of high accuracy and areas where the model's performance was less reliable. The classified RMSE accuracy map further categorizes the study area into high‐, moderate‐, and low‐accuracy zones, covering 15,848.26 km^2^, 6912.53 km^2^, and 353.23 km^2^, respectively Figure 12D. This classification clearly visualizes the model's performance across the region, indicating where predictions are most reliable.

An uncertainty analysis using Monte Carlo simulations was conducted to evaluate the reliability of the model's predictions. As visualized in Figure S3, predicted NDVI values are shown alongside their 95% confidence intervals, which represent the range of likely true values for each observation. Observations with high uncertainty, defined as having confidence interval widths exceeding 0.05, are highlighted in red. These observations indicate areas where the model may require refinement or where additional input data could enhance reliability. This analysis not only highlights the variability in predictions but also underscores the model's overall stability, with the majority of predictions exhibiting narrow confidence intervals and high reliability.

### 3.6 SHAP Analysis of NDVI Predictors in the Study Area and Plantation Sites

**FIGURE 13:**
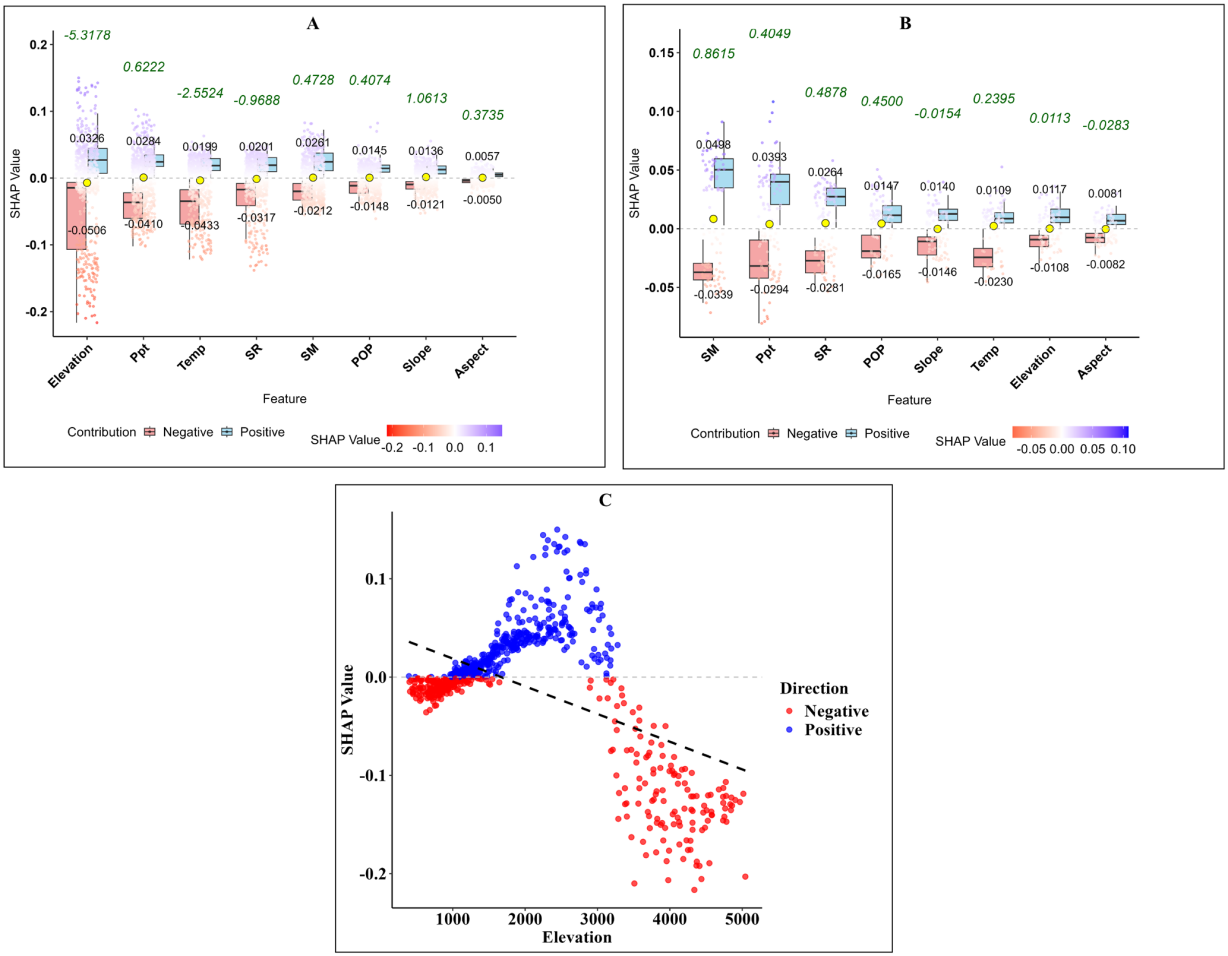
SHAP analysis of NDVI prediction model. (A) SHAP summary plot for the entire study area, illustrating the influence of various environmental and demographic variables on NDVI predictions. The magnitude and direction of SHAP values indicate the contribution of each variable, with positive values boosting predictions and negative values reducing them. (B) SHAP summary plot for the BTAP plantation sites, focusing on critical variables affecting NDVI predictions within these zones. Notably, soil moisture (SM) and precipitation (Ppt) emerge as dominant predictors. (C) Scatter plot depicting the relationship between SHAP values and elevation, revealing a nonlinear influence of elevation on NDVI predictions. Red dots represent negative contributions, while blue dots signify positive contributions, emphasizing the varying impact of elevation across the landscape.

A deeper understanding of the factors influencing NDVI predictions was achieved by employing SHAP to evaluate the relative importance of various environmental and demographic variables. The analysis was performed separately for the entire study area and the specific plantation sites associated with the BTAP. Elevation emerged as a key factor in the broader study area, consistently showing a strong negative influence on NDVI. This negative impact was particularly evident at elevations above 2000 m, where harsh environmental conditions, such as lower temperatures and shorter growing seasons, significantly limit vegetation growth. The average SHAP value for elevation was approximately −0.0506, highlighting its critical role in reducing NDVI at higher altitudes Figure 13C, where a decline in NDVI with increasing elevation is apparent. Precipitation showed a mixed influence, with SHAP values indicating positive and negative effects depending on specific regional conditions. The temperature had a more balanced impact, with average SHAP values around 0.0199, reflecting its varying influence on NDVI based on the local climate. On the other hand, soil moisture contributed positively to NDVI, with an average SHAP value of 0.0326, underscoring its importance in supporting vegetation across the study area Figure 13B.

In the plantation sites associated with the BTAP, the SHAP analysis identified soil moisture as the most influential variable, with a strong positive impact on NDVI. The average SHAP value for soil moisture was approximately 0.0498, indicating its vital role in promoting vegetation growth within these afforestation zones. Precipitation was also a significant factor, with an average SHAP value of 0.0398, emphasizing the importance of adequate rainfall for the success of plantation efforts. Additionally, SR and POP positively influenced NDVI, with average SHAP values of 0.0264 and 0.0147, respectively, reflecting their roles in vegetation recovery within these managed environments Figure 13A. Elevation in the plantation sites had a less noticeable but relevant impact, with an average SHAP value of 0.0117. Unlike the entire study area, elevation's influence here was more balanced, suggesting that the controlled conditions within the plantation sites helped mitigate some adverse effects typically associated with higher altitudes. This finding reflects the targeted management strategies implemented in these areas to counteract the challenges posed by elevation.

## 4 Discussion

### 4.1 BTAP Impact on Forest Cover and Land‐Use Dynamics

The results of this study demonstrate the significant positive impact of the BTAP in KPK on forest cover and overall land‐use dynamics between 2015 and 2023. Tree cover increased from 25.02% (5153.57 km^2^) in 2015 to 29.99% (6835.66 km^2^) in 2023, while barren land decreased from 20.64% (4251.24 km^2^) to 16.81% (3830.70 km^2^), indicating the success of the afforestation efforts in promoting ecological restoration. Similar afforestation projects have shown comparable outcomes; for instance, the “One Million‐Mu Plain Afforestation Project” in Beijing led to increased forest cover, though with varying greenness levels (Chen, Wang, and Jin 2021; Yu et al. 2018).

Additionally, the natural afforestation observed on abandoned agricultural lands in Russia and Belarus during the post‐Soviet period further underscores the positive impact of such initiatives on forest recovery and ecological restoration (Ershov et al. 2022). These comparative studies reinforce the success of the BTAP in enhancing forest cover and environmental health in the region. Using Sentinel‐2 imagery and the RF algorithm resulted in high‐accuracy LULC classifications, with an overall accuracy exceeding 85% for the years analyzed. Comparable studies have demonstrated similar efficacy in using RF for land‐cover classification. For instance, a method combining Landsat time series data with RF achieved an accuracy of 87% in predicting afforestation areas, underscoring the effectiveness of these techniques in large‐scale afforestation monitoring (Avci et al. 2023; Cavalli et al. 2023).

Additionally, research mapping forest changes in Guangdong Province using Landsat and PALSAR data reported classification accuracies between 75% and 85%, further validating the reliability of these methods for accurate LULC classification (Shen et al. 2019). These studies highlight the robustness of the approach used in this analysis. This allowed for a detailed analysis of changes over time, showcasing the effectiveness of BTAP in reversing deforestation and enhancing vegetation density. Similar outcomes were observed in a World Bank project in Nigeria and the Three‐North Afforestation Program in China, which significantly increased vegetation cover and reversed deforestation trends (Zhu et al. 2017). These studies affirm the positive impact of BTAP on ecological restoration.

### 4.2 Localized Impacts and Spatial Variability in Afforestation

Buffer analysis within the plantation zones revealed significant localized improvements, with tree cover in districts like Bajaur, Khyber, and Mohmand increasing by 90.28%, 78.79%, and 85.74%, respectively, from 2015 to 2023, like other successful afforestation projects that enhanced forest cover through targeted efforts (Ullah et al. 2021). This localized success is likely attributable to targeted afforestation strategies and favorable environmental conditions within these buffer zones, as shown in studies where strategic planning and species selection significantly improved outcomes (Qiu et al. 2019). However, the persistence of barren land in some districts, such as the 17.48% observed in 2023 within the plantation buffer zones, suggests that additional efforts are required to fully rehabilitate these areas, consistent with findings that ongoing interventions are necessary in arid regions (Liu et al. 2018; Tajik, Ayoubi, and Zeraatpisheh 2020). The results of the hotspot analysis, which showed an increase in high‐confidence hotspots from 36.76% in 2015 to 42.56% in 2023 and a corresponding decrease in high‐confidence cold spots from 28.03% to 21.34%, further confirm the positive vegetation trends resulting from BTAP interventions, aligning with studies demonstrating satisfactory spatial variability in afforestation outcomes (Wu et al. 2021).

### 4.3 Predictive Performance and Spatial Consistency in Afforestation

The machine‐learning‐based NDVI predictions using an ANN model yielded an *R* ^2^ value of 0.8556, with an RMSE of 0.0607, demonstrating strong predictive performance, like the high accuracies reported in studies using ANNs for environmental predictions (Celik et al. 2022; Emadi et al. 2020). SHAP analysis identified soil moisture (average SHAP value of 0.0498) and precipitation (average SHAP value of 0.0398) as the most influential variables driving vegetation growth, consistent with findings that emphasize the critical role of these factors in environmental modeling (Ren, Ling, and Wang 2023; Zhu et al. 2021). These findings suggest that successful afforestation in this region heavily depends on adequate water availability, aligning with previous research highlighting the importance of moisture for vegetation recovery (Akram et al. 2022; Fernández 2023; Otkin et al. 2019; Anees, Yang, and Mehmood 2024; Pan et al. 2023).

The identification of soil moisture and precipitation as key predictors offers actionable insights for afforestation management (Andreevich et al. 2020; Usoltsev et al. 2020; Shobairi et al. 2022; Usoltsev et al. 2022; Gong et al. 2024). For example, areas with low soil moisture could benefit from interventions such as soil moisture retention techniques, including mulching and soil amendments, to enhance vegetation survival and growth (Aslam et al. 2022). Additionally, aligning plantation schedules with periods of adequate rainfall can maximize the establishment success of young vegetation. These strategies, guided by SHAP findings, enable targeted resource allocation and improved decision making in afforestation initiatives, ultimately enhancing the resilience and effectiveness of such projects.

The spatial autocorrelation results, measured by Moran's *I* statistic, showed a consistent increase in positive spatial autocorrelation from 0.929 in 2015 to 0.933 in 2023 (*p* < 0.001), further supporting the observed clustering of NDVI values and indicating a sustained and nonrandom distribution of vegetation improvements over the study period. This finding aligns with studies on vegetation patterns in earthquake‐affected Southwest China, where Moran's *I* revealed significant clustering of damaged vegetation (Li et al. 2019). Another study using Moran's *I* to detect land‐cover change patterns in a large‐scale remote sensing imager demonstrates this statistic's effectiveness in identifying spatial clustering (Kiani et al. 2023; Self et al. 2023). This spatial consistency underscores the effectiveness of BTAP not only in isolated pockets but across the broader landscape of KPK, reflecting the broad‐scale impact of the project.

While the BTAP has achieved substantial gains in forest cover, the findings also highlight areas needing further attention, particularly in districts where barren land remains high or where tree cover improvements have been minimal. Similar challenges in reforestation, such as those related to seedling survival and land degradation, have been observed in other regions, underscoring the need for more intensive efforts in these areas (Flores et al. 2021; Román‐Dañobeytia et al. 2015). The persistence of barren land at 17.48% within the buffer zones suggests the need for more intensive reforestation efforts, a necessity echoed in studies emphasizing continuous forest management to address land‐use challenges and optimize reforestation outcomes (Song et al. 2023; Warner et al. 2022). Moreover, the transition of some areas from tree cover to shrubland and built‐up land, such as the 2.22 km^2^ transition to shrubland and 0.676 km^2^ to built‐up areas, suggests potential pressures from urbanization and land‐use change, which could undermine the long‐term success of the project. This pattern is consistent with research highlighting the negative impacts of urbanization on forest ecosystems and the challenges it poses to maintaining forest cover (Miroshnyk et al. 2022; Rai et al. 2023).

### 4.4 Limitations

This research adopted an all‐around approach by running hotspot and interannual analyses from 2000 to 2023 and considering the ground validation of the LULC classification for improved accuracy in the results. Yet, a few limitations exist. Although ground truth and site visits were done for rigorous verification of the LULC classification and effectiveness of BTAP, inherent challenges in remote sensing, such as resolution constraints and the potential influence of atmospheric conditions on satellite data, can never be fully resolved. Moreover, while the study period's length allows for capturing long‐term trends, the dynamism and complexity of most ecological processes may mean that some subtle or delayed effects from afforestation are not entirely captured within this study. The integration of ground‐truthing and site visits further strengthens the validity of results, reducing the limitations presented by this method.

### 4.5 Future Implications

Such future studies could benefit from further integrating remote sensing with ground‐based data collection, potentially through higher resolution or hyperspectral imagery, LiDAR, and UAV data. These technologies offer enhanced detail on vegetation dynamics, canopy structure, and biomass, which could complement Sentinel‐2 imagery to provide a more comprehensive understanding of afforestation impacts. Extending the study duration beyond 2023 and increasing the frequency of temporal observations would also help capture long‐term ecological changes and potential lag effects resulting from afforestation activities. Moreover, employing more sophisticated machine‐learning models could improve predictive accuracy, accommodating the complexities of ecological data and the interactions between environmental and socioeconomic variables. These advancements would support the development of sensitive, adaptive, and sustainable afforestation strategies, ensuring that projects like BTAP continue to make positive contributions to environmental restoration. Our findings demonstrate the effectiveness of large‐scale afforestation in landscape restoration, advocating for continuous monitoring and adaptive strategies to address emerging challenges. The integration of remote sensing and machine learning offers a robust framework for evaluating and guiding these efforts, providing data‐driven insights that are essential for sustainable land management and conservation.

## 5 Conclusion

The BTAP assessment conducted in this study demonstrates the effectiveness of large‐scale afforestation efforts in Pakistan using advanced remote sensing and machine‐learning techniques. Our approach enabled accurate LULC classification, revealing a substantial increase in tree cover from 25.02% in 2015 to 29.99% in 2023, and a corresponding decrease in barren land. Additionally, the hotspot analysis and spatial autocorrelation confirmed positive clustering in vegetation recovery, while the ANN model's predictive accuracy underscored the critical influence of soil moisture and precipitation on vegetation health. While these findings affirm the success of the BTAP, they also highlight the need for continuous monitoring and adaptive management to address challenges such as the persistence of barren land and transitions to shrubland or built‐up areas in some regions. We recommend that policymakers and project managers prioritize ongoing monitoring frameworks and adaptive strategies to ensure that afforestation efforts respond effectively to environmental changes. Engaging local communities in these efforts is essential, as their involvement can enhance land stewardship and contribute to sustainable management practices. Furthermore, implementing soil moisture retention techniques and optimizing planting schedules to align with precipitation patterns can support vegetation resilience in arid regions. By integrating data‐driven insights and fostering community partnerships, afforestation programs like the BTAP can achieve more sustainable and lasting ecological outcomes.

### 5.1 Recommendations

To enhance the effectiveness and sustainability of afforestation efforts, the following actions are recommended:

1. Intensify reforestation efforts in persistently barren areas by utilizing locally adapted species.
2. Implement enhanced monitoring and adaptive management strategies to ensure continuous sustainability.
3. Strengthen community engagement to align afforestation initiatives with socioeconomic needs.
4. Balance urbanization with ecological restoration through stringent land‐use planning.
5. To refine afforestation strategies, integrate higher resolution satellite data and advanced machine‐learning models in future research.

## Author Contributions

**Kaleem Mehmood:** conceptualization (equal), data curation (equal), formal analysis (equal), investigation (equal), methodology (equal), software (equal), validation (equal), visualization (equal), writing – original draft (equal), writing – review and editing (equal). **Shoaib Ahmad Anees:** conceptualization (equal), data curation (equal), formal analysis (equal), investigation (equal), methodology (equal), software (equal), supervision (equal), validation (equal), visualization (equal), writing – original draft (equal), writing – review and editing (equal). **Sultan Muhammad:** writing – review and editing (equal). **Fahad Shahzad:** writing – review and editing (equal). **Qijing Liu:** writing – review and editing (equal). **Waseem Razzaq Khan:** formal analysis (equal), investigation (equal), writing – review and editing (equal). **Mansour Shrahili:** writing – review and editing (equal). **Mohammad Javed Ansari:** writing – review and editing (equal). **Timothy Dube:** writing – review and editing (equal).

## Ethics Statement

The authors have nothing to report.

## Consent

The authors have nothing to report.

## Conflicts of Interest

The authors declare no conflicts of interest.

## Supporting information


Data S1.

## Data Availability

The authors confirm that the data links supporting the findings of this study are available within the article.
