# Supplementary material for: Machine Learning and Spatio Temporal Analysis for Assessing Ecological Impacts of the Billion Tree Afforestation Project
Source: Ecol Evol. 2025 Feb 19;15(2):e70736. doi: 10.1002/ece3.70736 (PMC11839268; doi:10.1002/ece3.70736)
Supplement: Supplementary file 1 — Data S1. [file ECE3-15-e70736-s001.docx]

**Supplementary Materials**


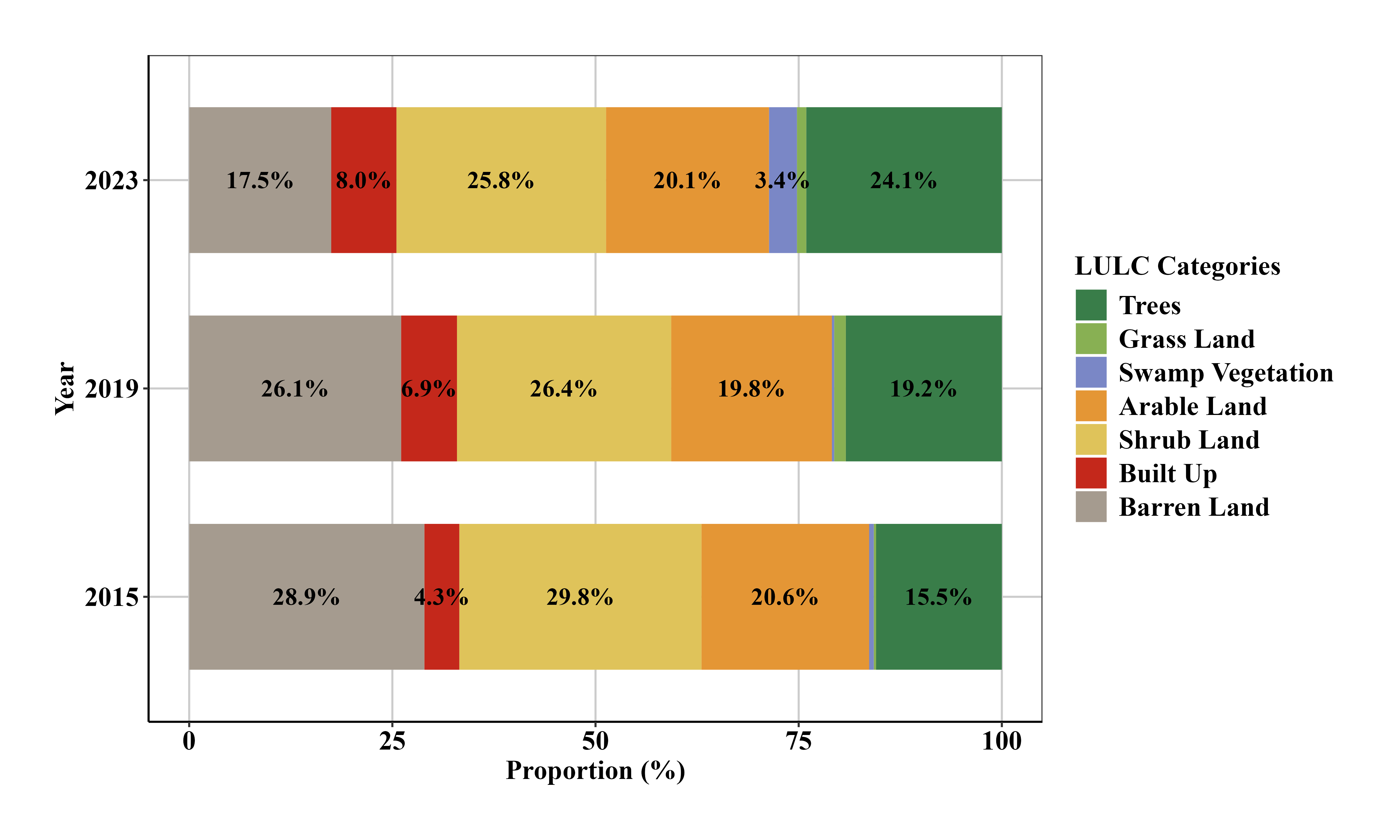


Figure S1: Buffer Analysis of Land Cover Comparison Across Districts and Plantation Sites from 2015 to 2023

**Correlation Plot (Supplementary Figure S2)**


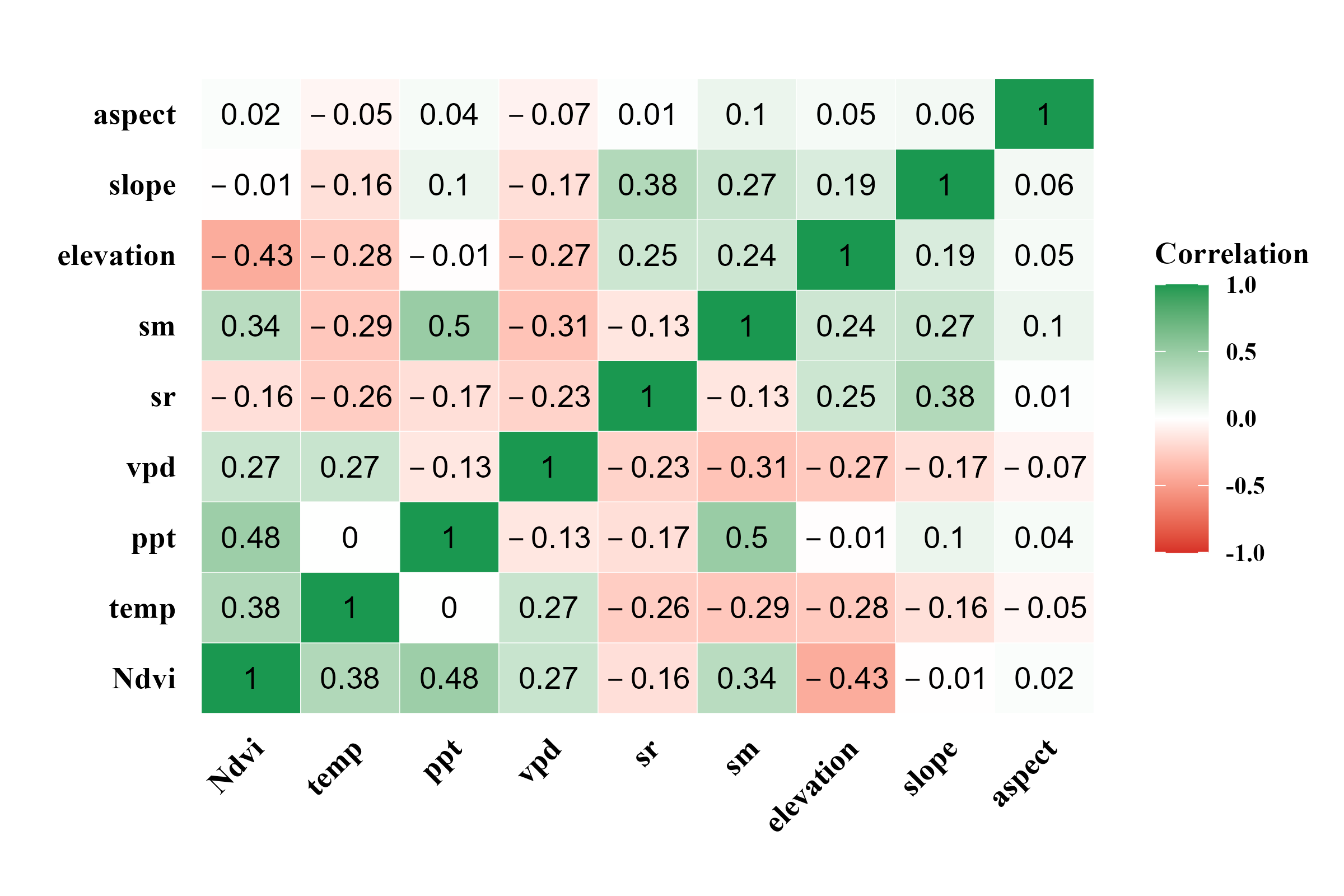


**Figure S2**: *Correlation matrix of predictor variables used in the ANN model for NDVI prediction. The plot displays the pairwise correlation coefficients (r values) between each variable, with values bolded and italicized for clarity. Colors range from red (negative correlation) to green (positive correlation), with white indicating no correlation. This matrix provides a visual assessment of multicollinearity among predictors.*

**VIF and Tolerance Table (Supplementary Table S1)**

**Table S1**: *Variance Inflation Factor (VIF) and Tolerance values for each predictor variable used in the ANN model for NDVI prediction. VIF values were calculated to assess multicollinearity among predictors, with a threshold of 10 indicating significant multicollinearity concerns. Tolerance is the inverse of VIF, providing an additional measure of each predictor’s independence. All VIF values were below the threshold, supporting the inclusion of each variable in the model.*

| **Predictor** | **VIF** | **Tolerance** |
| --- | --- | --- |
| **Temp** | 7.188973 | 0.13912 |
| **Ppt** | 1.554531 | 0.64328 |
| **Vpd** | 4.392184 | 0.22764 |
| **SR** | 9.640967 | 0.10372 |
| **SM** | 3.531995 | 0.28312 |
| **Elevation** | 1.249271 | 0.80046 |
| **Slope** | 1.809427 | 0.55266 |
| **Aspect** | 1.012686 | 0.98747 |

**Uncertainty Analysis of Predicted NDVI Values Using Monte Carlo Simulation**


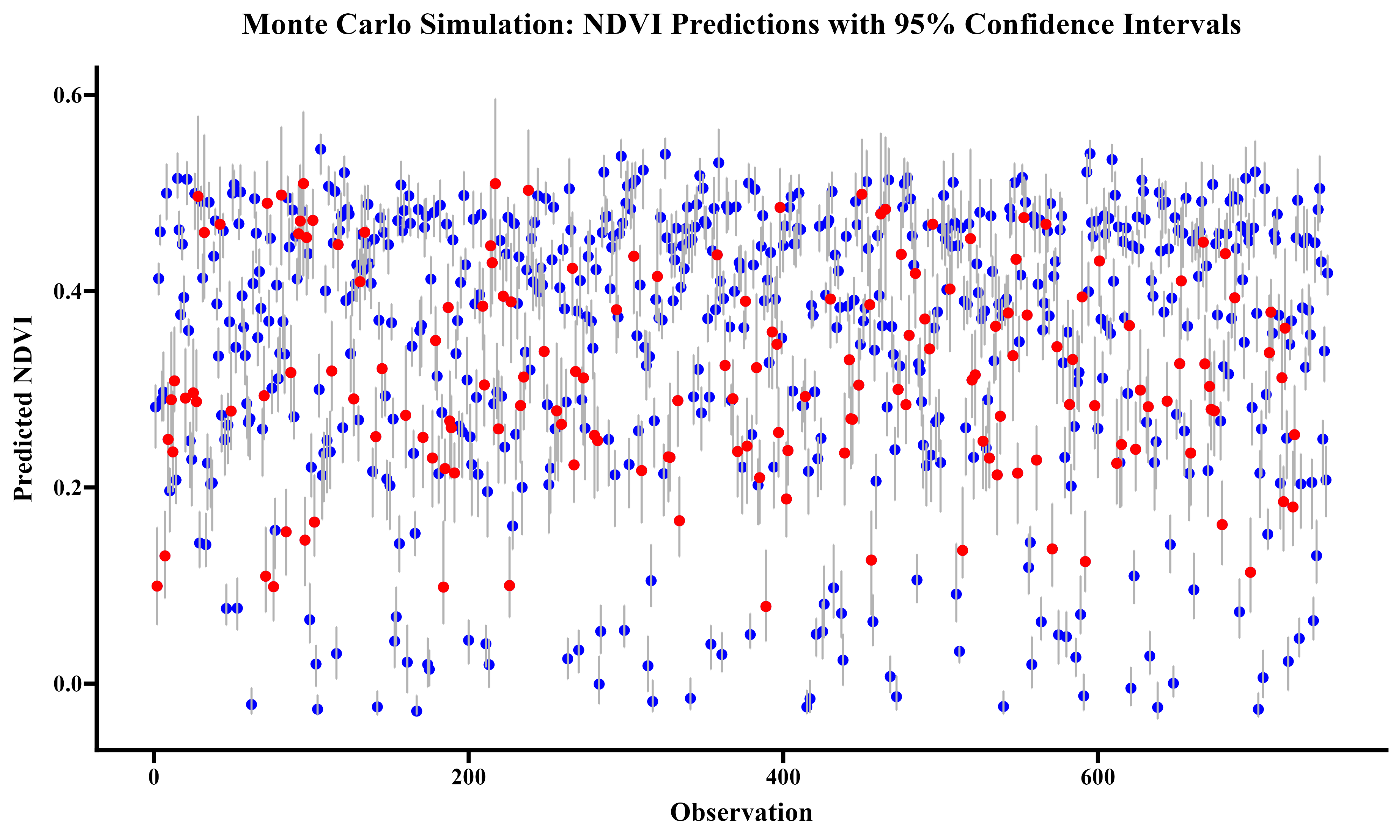


**Figure S3.** Uncertainty Analysis of Predicted NDVI Values Using Monte Carlo Simulation with 95% Confidence Intervals. This figure displays the predicted NDVI values across observations, with associated 95% confidence intervals represented by error bars. Red dots indicate observations with high uncertainty, highlighting areas where model predictions may be less reliable. This analysis provides insights into the stability of predictions and identifies areas where the model may benefit from further refinement or additional data.

**Validation of the ANN model on new plantation sites across three districts**


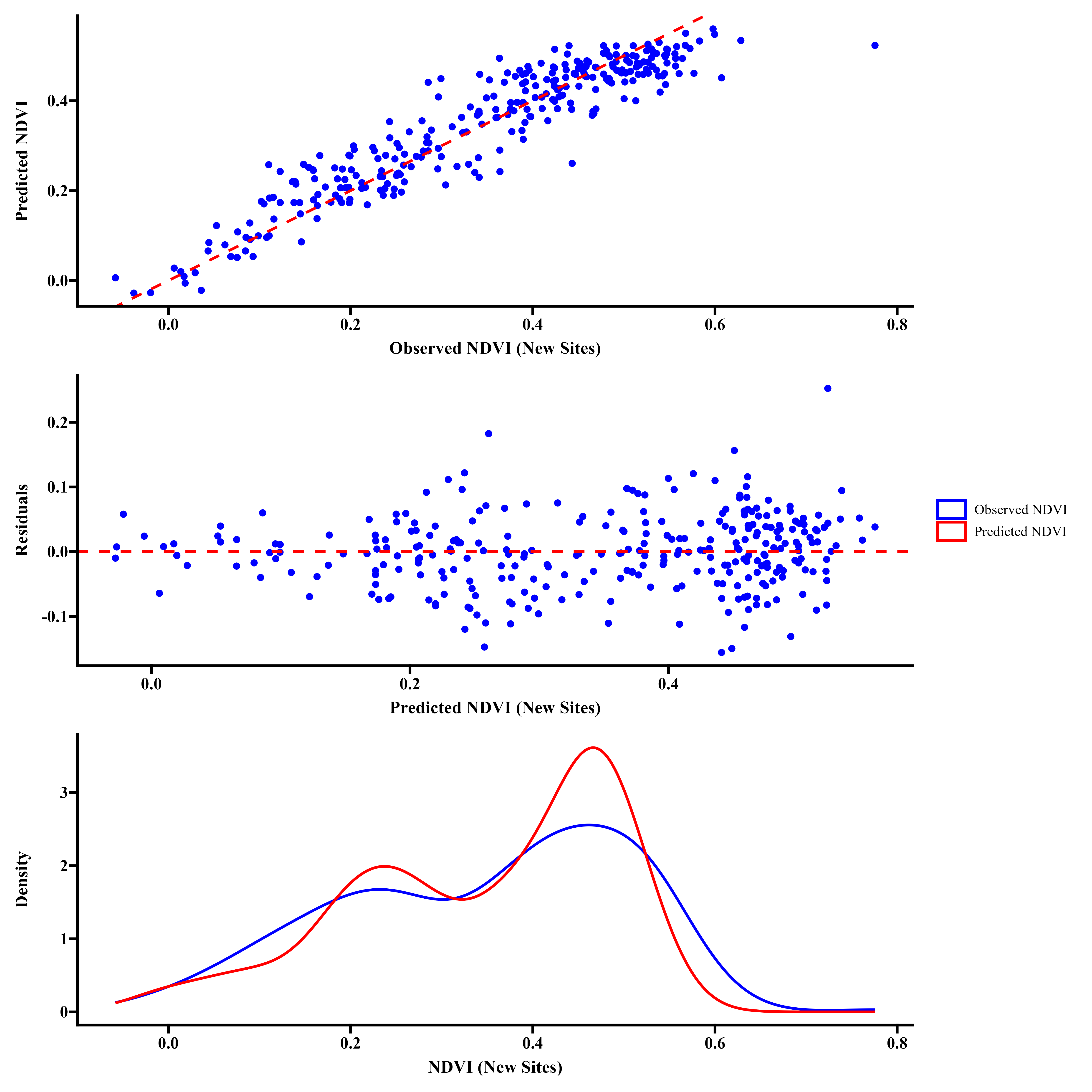


**Figure S4:** *Validation of the ANN model on new plantation sites across three districts. The first plot shows the scatterplot of predicted versus observed NDVI values, illustrating a strong agreement with minimal deviation along the 1:1 line. The second plot presents the residuals against predicted NDVI values, indicating random distribution without systematic bias. The third plot compares the density distributions of predicted and observed NDVI values, demonstrating the model's ability to capture the distribution of NDVI across the validation sites. These results reflect the model's robustness in generalizing unseen data from different locations.*
